# Inequitable Spatial and Temporal Patterns in the Distribution of Multiple Environmental Risks and Benefits in Metro Vancouver

**DOI:** 10.1029/2024GH001157

**Published:** 2024-12-19

**Authors:** Shuoqi Ren, Amanda Giang

**Affiliations:** ^1^ Institute for Resources Environment and Sustainability University of British Columbia Vancouver BC Canada; ^2^ Department of Mechanical Engineering University of British Columbia Vancouver BC Canada

**Keywords:** urban environmental quality, environmental inequity, cumulative exposure

## Abstract

The urban environment impacts residents' health and well‐being in many ways. Environmental benefits and risks may be interactively and inequitably distributed across different populations in cities, and these patterns may change over time. Here, we assess the spatial distribution of environmental risks and benefits in pairs, considering synergies and trade‐offs, in an illustrative metropolitan area (Metro Vancouver) in Canada in the years 2006 and 2016. We classify census dissemination areas as sweet, sour, risky, or medium spots based on relative exposures for six environmental combinations: Walkability and NO_2_; heat stress and NO_2_; vegetation coverage and NO_2_; vegetation coverage and heat stress; walkability and accessibility to natural recreational areas; and heat stress and accessibility to natural recreational areas. We evaluate whether different population groups are disproportionately exposed to lower environmental quality based on linear regressions and other metrics. We find that while performance for individual environmental variables improved over the decade, considering their combinations, sweet spots became sweeter and sour spots became sourer. Residents with high material and social deprivation and visible minorities were disproportionately exposed to lower environmental quality in both years for most of the environmental combinations. Further, we find that these inequities were not improving over time for all groups: for instance, South Asian residents in the region faced higher disproportionate burdens or diminished access to benefits in 2016, as compared to 2006. Given these findings, we suggest considerations of cumulative exposure in prioritizing areas for intervention, targeting the sour and risky spots persistently experienced by overburdened populations.

## Key Points

- Cumulative exposures to environmental risks and benefits have distinct spatial patterns compared to individual environmental factors
- Materially and socially deprived groups and visible minorities face greater exposures across risk‐benefit environmental combinations
- From 2006 to 2016, the performance of individual environmental factors improved, but South Asian residents experienced larger inequities

## 1 Introduction

Over 50% of the world's population lives in urban areas. In some countries, such as Canada, this share is even higher, at close to 74% (Statistics Canada, 2022; The World Bank, 2023). Urban environments can impact human health and well‐being in many ways, including through the prevalence of cardiovascular and respiratory diseases and the exacerbation of mental health challenges (Brulle & Pellow, 2006; Hankey & Marshall, 2017; James et al., 2017). Indeed, environmental risk factors caused 23.3% and 22.7% of total human mortality in 2004 and 2015 (Brusseau et al., 2019).

Urban environmental quality consists of a complex, interconnected mixture of factors. Some factors are health‐promoting, like high walkability and green spaces, while others present health risks, like air pollution and heat stress. Moreover, these different contributors do not impact human health and well‐being independently but are instead interconnected with potential synergies and trade‐offs (Morello‐Frosch et al., 2011; Nieuwenhuijsen, 2016). These interactions have the potential to either mitigate or amplify both adverse and beneficial health outcomes. For example, climate change has increased the frequency of extreme heat and wildfire smoke events, posing combined health risks. Co‐exposure to extreme heat and PM_2.5_ significantly increases cardiovascular and respiratory mortality risks (30% and 40%), according to a study in California (Rahman et al., 2022). Other examples of the interactive effects that environmental factors can have on human health and well‐being are summarized in Table 2. Given these interactions, assessments of environmental quality that account for multiple environmental factors and their synergies and trade‐offs can better capture the cumulative health impacts and then better support urban environmental planning and management (Z. Davis et al., 2022; Stossel et al., 2015).

The United Nations General Assembly and the Government of Canada have recognized that everyone has a right to a clean, healthy and sustainable environment with an emphasis on avoiding disproportionate adverse impacts on populations that may experience heightened vulnerability due to structural social, economic, and political factors or biological susceptibility (Environment and Climate Change Canada, 2023; UN. General Assembly, 2022). The cumulative impact of exposure to multiple environmental factors as well as social stressors (e.g., discrimination, social exclusion) and structural inequities can contribute to health disparities (Campbell, 2020; Morello‐Frosch et al., 2011). Researchers, regulators, and communities have consistently identified these cumulative impacts as a critical knowledge and environmental management gap (Gary et al., 2023; Racz & Rish, 2022; Su et al., 2009). Scholars and practitioners have highlighted different dimensions of environmental justice (EJ), including (but not limited to) distributive justice, procedural justice, and recognitional justice (Agyeman et al., 2016). Distributive justice, the focus of this study, is concerned with the fair distribution of environmental benefits and burdens; the analysis of distributive environmental injustice and its potential drivers can provide evidence for environmental decision‐making and support other types of EJ (Walker, 2010).

In Canada, populations that have been identified as potentially being disproportionately exposed to environmental risks (or limited in access to benefits), or experiencing higher vulnerability to resulting health impacts due to structural marginalization and biological susceptibility include Indigenous Peoples, Black Canadians and other People of Color, recent immigrants, migrant workers, those experiencing social and material depravation, children and older adults, and those with chronic health conditions—as well as intersections of these different identities (Giang et al., 2022; Pan‐Canadian Public Health Network, 2018; Waldron, 2022). For example, neighbourhoods with high walkability and active transportation plus low air pollution almost exclusively have high‐income residents, both in Metro Vancouver and Minneapolis in the U.S (Hankey et al., 2017; Marshall et al., 2009). This is similar for greenness and NO_2_ concentration, where in Toronto, Montreal, and Vancouver in Canada, postal codes with both high greenness and low NO_2_ concentration generally have lower material deprivation (Doiron et al., 2020). These studies also highlight the distinct patterns that emerge when considering multiple environmental factors in tandem, an important area for future study.

Environmental quality and the demographic distribution within urban areas are not static but evolve over time, potentially influenced by urban planning decisions such as the siting of industrial facilities, changes in land use, and zoning. These factors may thus be crucial drivers of the observed disproportionate distributional patterns of environmental quality (Estien et al., 2024; Lane et al., 2022; Mohai & Saha, 2015). For instance, research shows a historical discriminatory practice in the U.S., redlining, drives environmental quality disparities and residential segregation that results in EJ issues that persist today (Estien et al., 2024; Lane et al., 2022). Longitudinal analysis for distributional patterns of environmental quality could capture ongoing injustices and support exploring the drivers of the patterns.

In order to address the aforementioned gaps, this study aims to, for an illustrative metropolitan area (Metro Vancouver): (a) Characterize the spatial patterns of two‐dimensional environmental quality, considering combinations of 5 environmental factors (NO_2_ Concentration and heat stress as environmental burdens, and walkability, vegetation coverage, and accessibility to a park or water body as environmental benefits); (b) identify if there are disproportionate burdens in cumulative exposure to the two‐dimensional environmental quality for structurally marginalized and biologically susceptible populations, and (c) describe changes in environmental quality and distributional patterns between 2006 and 2016. Although the focus of this study is descriptive, in identifying patterns in space and time, it aims to support identifying potential drivers and priorities for action.

## 2 Methods and Data

### 2.1 Study Area and Resolution

This study focuses on urban areas (for more details on the definition of the urban area, please see Text S1 in Supporting Information S1) in the Metro Vancouver Regional District (MVRD), located in the Pacific coastal region of Southwestern Canada. Metro Vancouver consists of 21 municipalities, one electoral area, and one Treaty First Nation, and its residents reside on the shared territories of many Indigenous Peoples, including 10 First Nations: q̓ic̓əy̓̓ (Katzie), q̓ʷɑ:n̓ƛ̓ən̓ (Kwantlen), kʷikʷəƛ̓̓ əm (Kwikwetlem), máthxwi (Matsqui), xʷməθkʷəy̓əm (Musqueam), qiqéyt (Qayqayt), Semiahmoo, Sḵwx̱wú7mesh Úxwumixw (Squamish), scəw̓ aθən məsteyəxʷ (Tsawwassen), and səlilwətaɬ (Tsleil‐Waututh). It is one of the largest metropolitan areas in Canada, with a population of 2.6 million, according to the 2021 Canada Census (Statistics Canada, 2023). Metro Vancouver has high demographic diversity, for example, a significant population (54.4% of the total population) of visible minorities (in Canada, a census designation for non‐white and non‐Indigenous individuals) (Statistics Canada, 2023), with a clustered geospatial distribution.

Previous EJ studies have identified inequitable distributions of environmental burdens and benefits in Metro Vancouver (Frank et al., 2010; Henderson et al., 2022; Sax et al., 2022; Stieb et al., 2023), however, mostly focusing on single factors instead of cumulative exposures. Jurisdictions and health authorities in the region have identified environmental priority areas for continued improvement. For example, in both 2006 and 2016, annual NO_2_ concentrations in Metro Vancouver exceed WHO guideline levels (World Health Organization, 2021); and even though annual PM_2.5_ concentrations approach WHO air quality guideline levels (see Section 3.1), recent research suggests that there is no safe level of PM_2.5_ exposure for human health (Hoffmann et al., 2021). Air quality is also increasingly impacted by wildfires exacerbated by climate change (Yao et al., 2020). Metro Vancouver Regional District has published multiple phased regional growth strategies that involve goals like increasing community walkability and land use mix, improving air quality, and enhancing greenways and parks (Metro Vancouver, 2011). Policy suggestions for MVRD and regional planning for 2050 have also argued that addressing social equity issues is a key part of city planning for regional growth (Craig, 2021). Metro Vancouver therefore presents a useful illustrative case for exploring longitudinal environmental assessment and injustice characterization for our research objectives in cumulative exposures (Craig, 2021; Hoffmann et al., 2021; Statistics Canada, 2023; Yao et al., 2020).

Our study assesses changes over a 10‐year period by comparing two census years: 2006 and 2016. The resolution of this study for environmental quality assessment and distributional equity assessment is the Dissemination area (DA), which is the finest geographical unit for publicly available demographic data in Canada for Census data. Finer‐level environmental data are aggregated or converted to the DA level to match the demographic data. The aggregation method is the average of the finer‐resolution data in each DA, which is a proxy for the population weighting due to the approximately fixed population of around 19 households in each postal code and 400 to 700 population in each DA (Giang & Castellani, 2020). The numbers and boundaries of DA are slightly different in the two census years (maps for the study area are available in Figure S1 in Supporting Information S1). However, these differences do not impact the distributional equity assessment and comparison between years because the assessment metrics (i.e., linear regression coefficients and descriptive statistics) are calculated from demographic and environmental data within each year separately. Comparisons of spatial patterns between 2006 and 2016 for environmental and demographic data are only based on the DAs that exist for both years. This study assesses changes over time based on these two discrete time points; however, we note that the changes between 2006 and 2016 may not be monotonic.

### 2.2 Environmental Data

We focus on environmental factors for which there is evidence of significant impacts on human health and well‐being and for which there are publicly available data sources at fine spatial resolution for both study years. They include air pollution, walkability, vegetation coverage, accessibility to a park or water body, and heat stress. Table 1 summarizes the original sources of each data set, how they were accessed, and spatial and temporal resolution.

**Table 1:** The List of Environmental Variables, Metrics, Data Sources, Units, and Their Spatial and Temporal Resolutions

	Variables	Metrics	Unit	Spatial resolution	Years of data	Sources
Air Pollution	PM_2.5_	Ground‐level 3‐year annual average PM_2.5_ concentration (from the NASA MODIS, MISR, and SeaWIFS instruments with the GEOS‐Chem chemical transport model, calibrated to ground‐based observations using GWR)	μg/m^3^	Postal Code	Average of 2005, 2006, and 2007; average of 2015,2016, and 2017	Canadian Urban Environmental Health Research Consortium (CANUE)^a^
	Ozone	Ground‐level 3‐year annual average O_3_ concentration (from CHRONOS model and GEM‐MACH model)	ppb	Postal Code	Average of 2005, 2006, and 2007; average of 2013,2014, and 20132015	CANUE^b^ ^,^ ^c^ ^,^ ^d^ ^,^ ^e^
	SO_2_	Ground‐level 3‐year annual average SO_2_ concentration level (from Ozone Monitoring Instrument (OMI) satellite data using SO_2_ profiles from GEM‐MACH model)	ppb	Postal Code	Average of 2005,2006, and 2007; average of 2013,2014 and 20132015	CANUE^a^ ^,^ ^f^ ^,^ ^g^
	NO_2_	Annual average 3‐year NO_2_ concentration (from land use regression model using 2006 national air pollution surveillance (NAPS) monitoring data)	ppb	Postal Code	Average of 2004,2005,2006; average of 2014,2015,2016	CANUE^h^ ^.^ ^i^
Other Environmental Variables	Walkability	Canada Active Living Environment Index (Can‐ALE Index), including intersection density and dwelling density for 2006 and intersection density, dwelling density, and points of interest for 2016)	N/A	Postal Code	2006 and 2016	CANUE^j^
	Heat Stress	The average daily maximum apparent temperature/Humidex in July calculated from dew point and surface temperature	Celsius degree°C	1 km*1 km grid	2006 and 2016	Google Earth Engine (GEE): Daymet data set for surface temperature^k^ and ERA5 Daily Aggregates for dew points^l^
	Accessibility to recreational areas	Linear distance from a postal code to the nearest national, provincial, territorial, municipal, public and private level parks and recreation areas, regional parks, or water bodies (however, we do not have information on whether each water body is publicly accessible)	Meters (m)	Postal Code	2009 and 2014 for data from Canmap; 2006 and 2016 for data from Metro Vancouver	DMTI Spatial Inc. CanMap ‐ Park, Natural Recreational areas, and Water Bodies Boundry^m^ ^,^ ^n^ ^,^ ^o^; Metro Vancouver ‐ Regional Park Boundry^p^ and Historical Timeline^q^
	Vegetation Coverage	Annual mean value of NDVI (Normalized Difference Vegetation Index) from the USGS Landsat 5 and Landsat 8 satellites accessed from GEE	NA	Postal Code	2006 and 2016	CANUE^r^ ^,^ ^s^ ^,^ ^t^ ^,^ ^u^ ^,^ ^v^

*Note.* Years of data are chosen based on data availability for 2006 and 2016 to match demographic data. The concentrations of air pollutants are 3‐year annual averages around the two census years to account for interannual variability.

^a^ Hammer et al. (2020).

^b^ Environment and Climate Change Canada (2017a).

^c^ Environment and Climate Change Canada (2017b).

^d^ Robichaud et al. (2016).

^e^ Robichaud & Ménard (2014).

^f^ Environment and Climate Change Canada (2017c).

^g^ McLinden et al. (2014).

^h^ Hystad et al. (2011).

^i^ Weichenthal et al. (2017).

^j^ Ross et al., 2018.

^k^ Thornton et al. (2022).

^l^ Copernicus Climate Change Service (C3S) (2017).

^m^ DMTI Spatial Inc (2010).

^n^ DMTI Spatial Inc. (2014).

^o^ DMTI Spatial Inc, (2015).

^p^ Metro Metro Vancouver (2021a).

^q^ Metro Metro Vancouver (2021b).

^r^ Google Earth Engine Explorer (2017b).

^s^ Google Earth Engine Explorer (2017a).

^t^ Gorelick et al. (2017).

^u^ USGS (2017b).

^v^ USGS (2017a).

*Air pollution.* There is a large body of research indicates that long and short‐term exposures to air pollutants such as nitrogen dioxide (NO_2_), ground‐level annual fine particulate matter (PM_2.5_), ground‐level ozone (O_3_), and sulfur dioxide (SO_2_), have adverse health effects and can increase the risk of respiratory disease and all‐cause and respiratory mortality (World Health Organization, 2021). In this study, we focus on NO_2_ as an illustrative air pollution exposure. NO_2_ is one of the major components of traffic‐related air pollution (TRAP), which has been linked to air pollution exposure disparities in past studies (Clark et al., 2017; Guo et al., 2020), including in Canada (Pinault et al., 2016). However, we note that NO_2_ exposure does not represent all kinds of exposure to air pollution, as other air pollutants, including O_3_, SO_2_, and PM_2.5_, can have distinct sources, chemistry, and transport mechanisms. We briefly discuss spatial and temporal patterns for these other pollutants in 3.1 but focus analysis on NO_2_.

*Walkability.* To capture walkability, we use the Active Living Environment Index (ALE index), which measures the degree to which the built environment in a neighborhood supports the active mobility of residents (Ross et al., 2018). Active mobility is linked to multiple health benefits, such as lower incidences of type 2 diabetes, high body mass index, and cardiovascular diseases (Mueller et al., 2015). However, a high level of walkability can increase residents' exposure to air pollution and lead to adverse health effects (Hankey et al., 2012; Howell et al., 2019; James et al., 2017; Marshall et al., 2009).

*Vegetation coverage.* Vegetation coverage is measured by the Normalized Difference Vegetation Index (NDVI), which ranges from −1 to 1 (−1 represents water surface and 1 represents dense vegetation). Increasing vegetation coverage can positively impact human health directly or indirectly, for example, by decreasing all‐cause mortality and reducing the association between air pollution and mortality (Brochu et al., 2022; Crouse et al., 2019).

*Heat stress exposure.* We use the average daily maximum apparent temperature in July 2006 and 2016 to operationalize heat stress. Apparent temperature (Humidex) reflects human perceived temperature, which is the most direct indicator of human heat exposure and heat‐related mortality (Ho et al., 2016; Zhang et al., 2014). Apparent temperature takes relative humidity into consideration; it is calculated from the dew point and air temperature extracted as raster data from Google Earth Engine (GEE) using the Humidex equation (Ho et al., 2016). High levels of humidity increase health risks when air temperature is greater than skin temperature by limiting evaporative cooling (R. E. Davis et al., 2016). Apparent temperature has been robustly correlated to heat‐related mortality (Zhang et al., 2014). However, there are other dimensions that could be concerned when measuring heat stress which are not captured in this metric; for example, the multi‐day duration of heat events (Gasparrini & Armstrong, 2011).

*Accessibility to a natural recreational area.* The accessibility to a park or water body is calculated from the Euclidean distance of the boundary of the nearest public park (including natural recreational areas, see Table 1) or a water body to a postal code. In addition to vegetation coverage, parks and water bodies can provide mental health and well‐being benefits and other benefits brought by physical recreational activities, which may not be substituted by simple functional vegetation like street trees or lawns (Gascon et al., 2015; Nguyen et al., 2021; White et al., 2020). Alternatively, there are other metrics in the literature, like considering the actual road distance and the transportation availability (Maroko et al., 2009; Park et al., 2021).

### 2.3 Demographic Data

Socio‐demographic data are extracted at the DA level from 2006 to 2016 Canadian Census data, accessed through the Canadian Census analyzer (Canadian Census Analyser at CHASS, 2021). Based on previous EJ research, we consider the following census variables that have been linked to increased biological susceptibility or structural vulnerability to environmental health risks in Canada (Giang et al., 2022; Pan‐Canadian Public Health Network, 2018; Waldron, 2022): racialization and ethnicity (“Visible Minority”), Indigenous identity (“Aboriginal Identity”), educational attainment, recent immigrant status (arrival within 5 years before the Census year), low‐income status (based on the low‐income cut‐off, LICO), age (population of 0–14 and 65+), and employment status. Statistics Canada classifies Canadians' racialization and ethnicity by dividing the total population into “visible minorities” (South Asian, Chinese, Black, Filipino, Latin American, Arab, Southeast Asian, West Asian, Korean, Japanese, visible minorities not included elsewhere (visible minority n.i.e), and multiple visible minorities), Indigenous, and White. We also include composite social and material deprivation indices at the DA level for both years from the Institut national de santé publique du Québec (INSPQ), that bring together multiple socio‐economic indicators (Gamache et al., 2019). For example, a sub‐indicator for social deprivation is living alone; a sub‐indicator for material deprivation is access to adequate housing (Silva et al., 2024). More details on demographic variables are included in Text S2 in Supporting Information S1.

### 2.4 Environmental Quality Assessment Method

To capture the interactions between environmental variables, we conduct a two‐dimensional environmental assessment to identify the environmental “sweet,” “sour,” “risky,” and “medium” spots in urban areas in Metro Vancouver, which are referred to as sweet and sour spots analysis in the following content (Doiron et al., 2020). “Sweet spots” refer to the DAs in which both environmental variables have favorable performance in terms of impacts on human health: for example, low air pollutant concentration and high walkability. “Sour spots” indicate environments that are unfavorable in terms of both variables: for example, high air pollutant concentration and low walkability. We define ‘risky spots' as scenarios where the positive impact of one environmental variable on human health is not only offset by the adverse effects of another but may potentially magnify those adverse effects. For example, areas characterized by high air pollution yet high walkability fall under this category. In such instances, the health risks associated with air pollution could undermine the benefits derived from walkability (Frank et al., 2010; Howell et al., 2019; Marshall et al., 2009). “Medium spots” are the DAs that are not identified as sweet, sour, or risky spots, representing a category of DA with moderate environmental performance. For example, for the combinations other than walkability and NO_2_, the DAs where one variable has high performance while the other has low performance but there is no antagonistic interaction or two variables both have middle‐level performance. See Figure S2 in Supporting Information S1 for an illustrative figure for the classification of sweet, sour, and risky spots. Sweet and sour spot analysis is a powerful tool for characterizing the cumulative exposure to two to three variables in the environment considering their interactions (Doiron et al., 2020). Furthermore, it can inform decision‐making to prioritize neighborhoods for intervention. Table 2 shows the 6 main environmental combinations explored in this study, alongside the rationales for selecting these specific combinations. It also details the criteria for identifying sweet, sour, and risky spots within each combination.

**Table 2:** The Determinations and Rationales of Sour, Risky, Medium, and Sweet Spots for Environmental Combinations

No.	Environmental combinations	Sour/1	Risky/2	Medium/3	Sweet/4	Synergies or trade‐offs in health effects
1	**Walkability and NO**_**2**_	Class 1 and Class 1	Class 4 and Class 1	Other Class combinations	Class 4 and Class 4	High walkability can magnify the health risks brought by high air pollutant concentration due to increased exposure during physical activities^a^ ^,^ ^b^ ^,^ ^c^
2	**Heat Stress and NO**_**2**_	Class 1 and Class 1/Class 1 and Class 2/Class 2 and Class 1	NA	Other Class combinations	Class 4 and Class 4	Co‐exposure to heat stress and air pollution could lead to larger adverse health effects^d^
3	**Vegetation Coverage and NO**_**2**_	Class 1 and Class 1	NA	Other Class combinations	Class 4 and Class 4/Class 3 and Class 4/Class 4 and Class 3	Larger coverage of vegetation can mitigate the impacts of air pollution^e^
4	**Vegetation Coverage and Heat Stress**	Class 1 and Class 1	NA	Other Class combinations	Class 4 and Class 4/Class 3 and Class 4/Class 4 and Class 3	Larger coverage of vegetation can mitigate the impacts of heat stress, for example, providing shading and cooling effects^f^ ^,^ ^g^
5	**Walkability and Accessibility to a Park or Waterbody**	Class 1 and Class 1/Class 1 and Class 2/Class 2 and Class 1	NA	Other Class combinations	Class 4 and Class 4/Class 3 and Class 4/Class 4 and Class 3	Low walkability can limit people's accessibility to a park or water body; high walkability can support higher access to a park or water body^h^ ^,^ ^i^
6	**Heat Stress and Accessibility to a Park or Waterbody**	Class 1 and Class 1/Class 1 and Class 2/Class 2 and Class 1	NA	Other Class combinations	Class 4 and Class 4	The adverse effects of heat stress would be increased when people have very limited access to a park or water body^j^

*Note.* Classes 1 to 4 represent performance from unfavorable to favorable for each environmental variable.

^a^ Frank et al. (2010).

^b^ Howell et al. (2019).

^c^ Marshall et al. (2009).

^d^ Rahman et al. (2022).

^e^ Franchini & Mannucci (2018).

^f^ Lafortezza et al. (2009).

^g^ Zupancic et al., 2015.

^h^ Grow et al. (2008).

^i^ Richardson et al. (2020).

^j^ Lafortezza et al. (2009).

We apply two methods, even breaks and population quartiles, to determine the performance of each variable into four classes. The four classes rank from 1 to 4, indicating the most unfavorable to most favorable performance of each environmental variable. The even breaks method aims to equally divide the DAs into four classes based on the minimum and maximum values of each environmental variable. The other method is to divide the DAs based on the population quartiles of each environmental variable. While determining classes from even breaks captures the favorable and unfavorable environmental performance based on absolute values, the population quartile method can ensure each class contains the same number of DAs even when the distribution of the variable is highly skewed with extreme values. We present findings from the quartile method in the Results section, discuss differences between the methods in Text S3 in Supporting Information S1, and present findings from the even breaks method from Figure S29 to Figure S30 in Supporting Information S1.

As shown in Table 2, sweet, sour, risky, and medium spots are defined based on how a DA performs for two environmental variables with considerations of the synergies and trade‐offs between them. For example, high walkability can support people's access to nearby parks or water bodies (Lafortezza et al., 2009); as a result, the health benefits are magnified. Therefore, we define the combination of walkability and access to a park or water body as a sweet spot even if one of the variables is in class 3.

### 2.5 Measures of Relative Environmental Distributional Inequity

Based on previous definitions (Anguelovski, 2016; Brulle & Pellow, 2006; Giang et al., 2022; Maguire & Sheriff, 2011), here we characterize distributional environmental injustice, interchangeably used with environmental inequity or inequitable exposure, as when historically and systemically marginalized populations, and/or those that may experience social risk factors for health in the Canadian context, experience disparities in exposure/access that may be health‐harming. We note that due to the relative metric used in this study, estimates of distributional inequity are within the context of Metro Vancouver for the years 2006 and 2016 only. Relative metrics can identify populations that face higher risks in a specific region. We also discuss findings with respect to absolute environmental quality and exposure levels and relevant health and well‐being benchmarks, where available.

We quantify distributional inequity for each demographic group in two ways: (a). Comparison of descriptive statistics; (b). Simple linear regression analysis. In terms of descriptive statistics, we define inequitable exposure as higher percentages of structurally marginalized or biologically susceptible populations in the sour and risky spots than in medium and sweet spots, shown in violin and box plots. The percentages of each population in sweet, sour, risky, and medium spots are summarized statistically via median, interquartile range (IQR), maximum and minimum (excluding outliers), mean, and probability density.

We perform simple linear regression using the Ordinary Least Squares method to quantitatively explore the linear relationship between the environmental spots category (coded as integer) and demographic distribution (percentage of the demographic variable in each DA). This approach yields a simple summary metric (linear regression coefficient) that facilitates comparison across a large number of environmental combinations and demographic groups and captures distribution across the four different spot types. Sweet spots, medium, risky, and sour spots are coded with 4, 3, 2, and 1, respectively, to numerically represent their ordinal categories, representing performance from favorable to unfavorable to health and well‐being. It is important to note that the interval between each integer does not represent the same magnitude of difference in health impacts. For the purpose of comparing the relative inequity level across demographic groups and environmental combinations, the integers from 1 to 4 are used for the simple linear regression for all environmental combinations even if there is no risky spot for some combinations. We also do a version of regression analysis that eliminates risky spots as a sensitivity analysis, and the results are largely robust, particularly in terms of identifying inequity and comparing the relative inequity level across groups for each year (focusing on the combinations without risky spots). Differences are discussed in more detail in Figures S31, S32 and Text S4 in Supporting Information S1.

As summarized in Table S8 in Supporting Information S1, here, we define a pattern of distributional inequity as when the percentage of a structurally or biologically vulnerable demographic group in a DA is negatively correlated with environmental quality (by sweet‐and‐sour spot category), resulting in a negative regression coefficient. The absolute value of the regression coefficient represents the magnitude of the slope, indicating the relative level of distributional inequity. Given our definition of distributional inequity‐‐areas with more vulnerable populations tending to be sour or risky spots‐‐the regression coefficients are only meant to represent the relative magnitude and direction of the linear relationship, rather than modeling the physical relationships between them for other areas or suggesting any causal relationships. We discuss other possible regression methods and expand on the implications of this approach in the Discussion section.

We also quantitatively evaluate whether patterns of environmental inequity change between the two study years, 2006 and 2016, by comparing the magnitude and direction of the slope of the linear fit for the same environmental category and demographic combination in two years, with criteria summarized in Table 3.

**Table 3:** Criteria Used to Identify Changing Environmental Justice Patterns Between 2006 and 2016 Based on the Regression Coefficient

		Environmental inequity pattern in each year	
		2006	2016
**Environmental inequity changing patterns**	**Improved**	Inequity identified	Not significant/Not identified
		Inequity was identified for both years ‐ the regression coefficient increased ↑ for marginalized and vulnerable groups	
	**Worsen**	Not significant/Not identified	Inequity identified
		Inequity identified for both years—the regression coefficient decreased ↓ for marginalized and vulnerable groups	
	**NA**	No inequity identified/Not significant in both year	

## 3 Results

### 3.1 Environmental Quality Characterization

The selected environmental variables show distinct patterns in terms of the range of absolute values and spatial distribution in the study region. Some variables have significant spatial heterogeneity, including NO_2_, accessibility to natural recreational areas, vegetation coverage, and walkability. For example, the area with the lowest annual NO_2_ concentration in 2016 was below the WHO annual air quality guideline level (4 ppb compared to 5.32 ppb), while the area with the highest annual concentration was close to six times the guideline level (World Health Organization, 2021). The shortest average Euclidean distance from a postal code to the nearest boundary of a park or a water body is 0 m while the longest distance is over 2 km. NDVI ranges from 0.03 (indicating sparse vegetation) to 0.64 (indicating dense vegetation). Other variables have lower absolute ranges. For example, the difference in apparent temperature was around 3°C in both years with a minimum temperature of 24°C in 2016 and 25.3°C in 2006. The WHO guideline for PM_2.5_ is 5 μg/m^3^, and the lowest annual concentrations of PM_2.5_ in both years were around this level (5.74 μg/m^3^ and 4.04 μg/m^3^). The highest concentrations were not significantly different as well (8.17 μg/m^3^ and 7.11 μg/m^3^, lower than the highest suggested interim target, 10 μg/m^3^) (World Health Organization, 2021). Similarly, annual O_3_ and SO_2_ concentrations both had more limited ranges and annual SO_2_ concentrations in both years were lower than current Metro Vancouver's ambient air quality objective (5 ppb) (Doerksen et al., 2020). For more details, see Tables S1–S4 in Supporting Information S1 for descriptive statistics of environmental and demographic variables, Tables S5 and S6 in Supporting Information S1 for population‐weighted average of environmental variables in different demographic groups, and Figures S3–S10 in Supporting Information S1 for spatial distribution maps for environmental variables in the year 2006.

The ranges of environmental variables in each combination are shown in Table S7 in Supporting Information S1. For example, for the combination of vegetation coverage and NO_2_ concentration in the year 2006, the average NO_2_ concentration is 11.61 ppb in sweet spots and 28.73 ppb in sour spots. The average NDVI is 0.49 in the sweet spots and 0.17 in the sour spots. Compared to the WHO annual AQG for NO_2_ (5.32 ppb), although the average performance of sweet spots also does not meet the guideline, the sour spots have a much higher concentration level. In 2016, the average NO_2_ concentration is 8.5 ppb in sweet spots and 23.28 ppb in sour spots. Both the differences between sweet and sour spots and absolute values improved; this pattern applies to most of the combinations.

Table S9 in Supporting Information S1 shows the proportions of sweet, sour, risky, and medium spots for each environmental combination. For some environmental combinations, such as walkability and accessibility to a park or waterbody, as well as heat stress and NO_2_, a large number of DAs show unfavorable performance (i.e., fall in the lowest population quartile) for both factors. As a result, close to a quarter of DAs are classified as sour spots (around 24% and 22%, respectively, for the two combinations for both years). Similarly, many DAs showing favorable performance for two factors at the same time would yield a high number of sweet spots. However, it is noteworthy that, with the exception of combinations involving vegetation coverage, the count of sweet spots is significantly lower than that of sour spots for both years. This suggests that DAs with favorable environmental conditions, when considering two factors, are less prevalent.

Between 2006 and 2016, all variables, except vegetation coverage, improved in population‐weighted average performance, and most environmental combinations have better performance and a decreasing proportion of sour spots in 2016. However, some environmental combinations have an increasing proportion of sour spots: for instance, a 2.18% increase in vegetation coverage and heat stress. A decrease in both sweet spots and sour spots indicates more DAs are categorized as medium performance in 2016, like heat stress and NO_2_. In general, environmental quality improved in 2016 compared to what we observed in 2006 and increased environmental benefits (e.g., walkability) in both sweet and sour spots.

**Figure 1:**
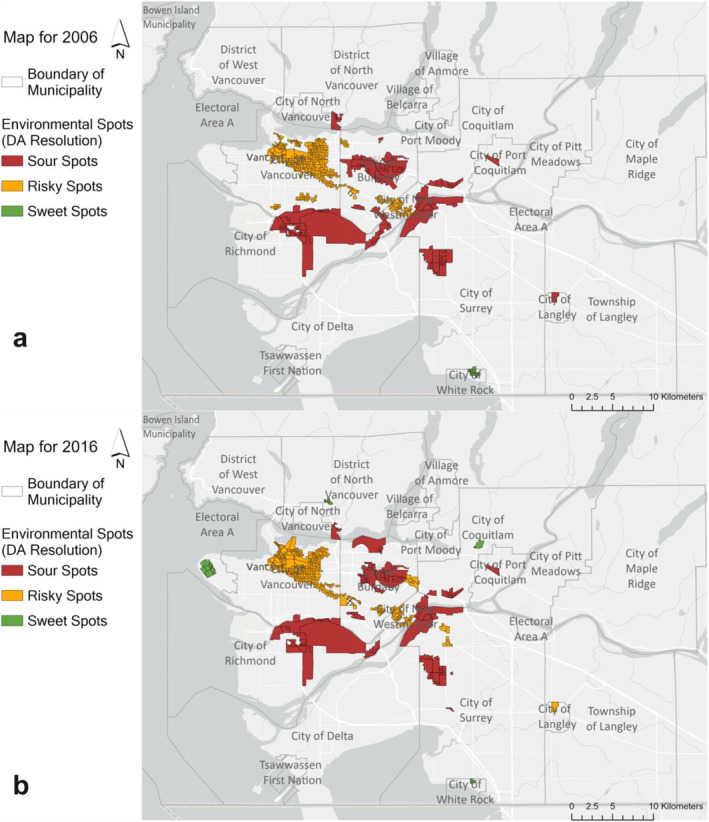
Spatial Distribution of NO_2_ and Walkability in 2006 (Figure. a) and 2016 (Figure. b). DAs in Red are Sour Spots, DAs in Orange are Risky Spots, and DAs in Green are Sweet Spots.

When we consider different environmental combinations, they demonstrate distinct spatial patterns; for example, some (e.g., vegetation coverage and NO_2_ as well as vegetation coverage and heat stress) have a large number of sweet spots that are widely spread while others (e.g., walkability and NO_2_) yield only sour and risky spots. At the same time, these impacts are not evenly distributed across Metro Vancouver. Figure 1 shows an illustrative example, focusing on NO_2_ concentration and walkability. NO_2_ is a traffic‐related air pollutant, and its spatial patterns are largely driven by major roads with high traffic volume. As a result, the risky spots with high walkability and high NO_2_ concentrations are clustered along major arterials in the City of Vancouver and other municipalities centers. Although sweet spots emerged in 2016, the spatial patterns did not change substantially. These risky spots may go unnoticed if we only focus on the distribution of walkable communities; although they have high performance on walkability, these areas could have unexpected risks to human health when NO_2_ exposure is considered, highlighting the need to consider cumulative exposure patterns. Other environmental combinations are shown in Figures S11–S15 in Supporting Information S1.

**Figure 2:**
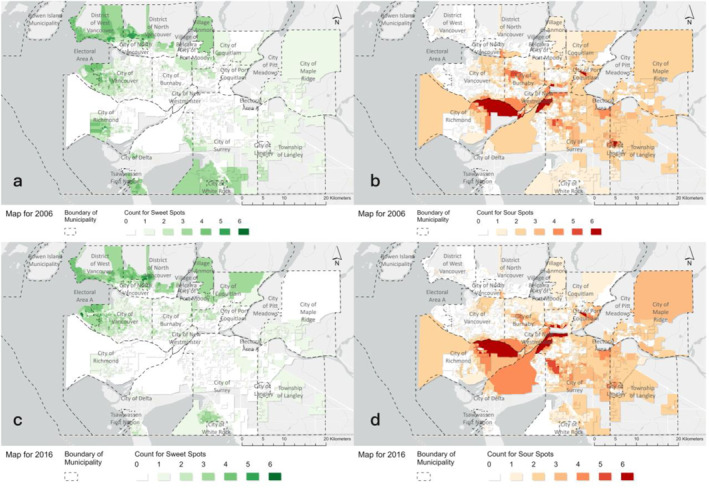
Sweet and sour heat maps for 2006 and 2016 indicating the total number that each Dissemination area recognized as sweet or sour spots in 6 environmental combinations. Sweet spot heat map for 2006 (Figure. a), sour spot heat map for 2006 (Figure. b), sweet spot heat map for 2016 (Figure. c), and sour spot map for 2016 (Figure. d).

Looking across all environmental combinations, we identify some regions of Metro Vancouver with high proportions of sweet or sour spots. Figure 2 shows the aggregate performance of each DA for the 6 environmental combinations, as the number of sweet spots and sour spots for that DA. The spatial patterns in 2006 and 2016 are highly similar. Across both years, the eastern and northern parts of Metro Vancouver, including the cities of Vancouver, West Vancouver and North Vancouver, have a significant proportion of sweet spots. In contrast, the majority of DAs in other municipalities are identified as sour or medium spots for many combinations. The most noticeable differences between the 2 years include the decreasing counts of sweet spots and increasing counts of sour spots in the southern part of Metro Vancouver (e.g., the city of Richmond and city of Surrey), which are areas with high proportions of visible minorities.

### 3.2 Distributional Environmental Inequity Quantification

**Figure 3:**
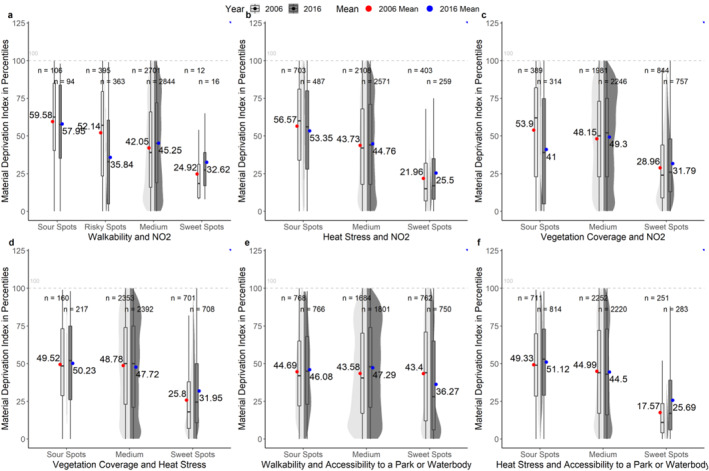
Statistical Summary of Material Deprivation in Different Environmental Spots. The mean values of material deprivation index in different environmental spots are labeled in blue for 2006 and in red for 2016. The numbers of DAs in each category are labeled at the top of the box plots; for example, *n* = 106 indicates there are 106 DAs classified as sour spots for the combination of walkability and NO_2_ in the year 2006. Note: The *X*‐axis for walkability and NO_2_ is different from other environmental combinations, given the existence of risky spots.

Figure 3 summarizes how the residential locations of different demographic groups are distributed across sweet, sour, risky, and medium spots through violin and box plots, using composite material deprivation as an illustrative example (for other demographic groups, see, from Figure S16 to Figure S26 in Supporting Information S1). As shown in Figure 3, for most combinations, the mean deprivation index in sour and risky spots is higher than in sweet spots in both years, indicating inequitable distributions of environmental risks and benefits. As an example, the mean material deprivation index in sour, risky, medium, and sweet spots for walkability and NO_2_ in 2006 are 59.58, 52.14, 42.05, and 24.92, respectively. Similarly, for vegetation coverage and heat stress, the mean material deprivation index in sour, medium, and sweet spots are 49.52, 48.78, and 25.80 in the year 2006. Although there is no large difference between the sour and medium spots, there is a dramatic drop in the material deprivation index in the sweet spots, highlighting the inequitable distribution of environmental benefits and risks.

**Figure 4:**
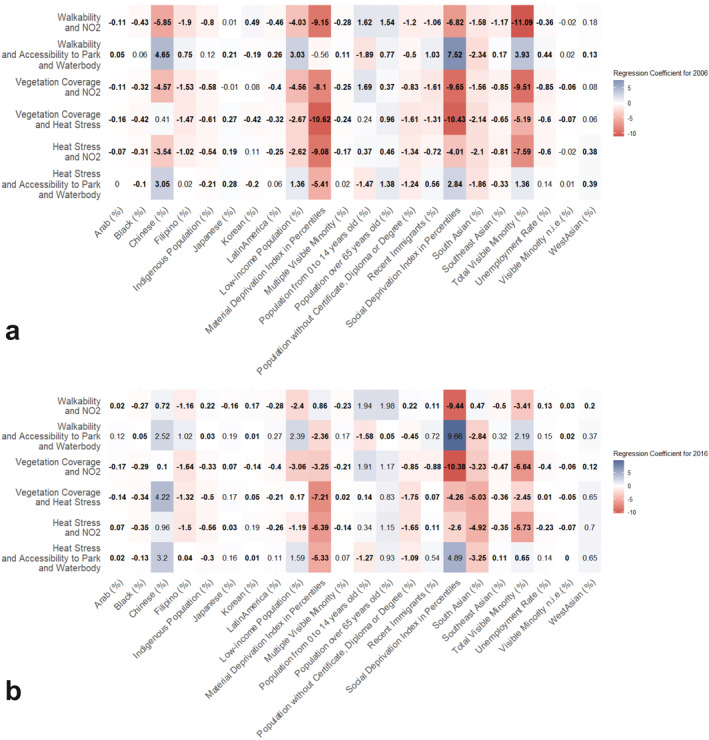
Heat Map for Regression Coefficients among Demographic Groups and Environmental Combinations in 2006 (a) and 2016 (b) (Quartile Method). We define inequity as a negative linear relationship between the marginalized demographic group percentage and the environmental category, indicated by a negative regression coefficient in red. Blue indicates no inequity identified for the given marginalized groups resulting from the positive linear relationship between demographic percentage and environmental category. Numbers in bold indicate that the coefficients are statistically significant; numbers that are not in bold (regular font) suggest no statistically significant inequity was identified, indicated by regression coefficient with a p‐value exceeding 0.05.

To summarize these linear relationships quantitatively, we use simple linear regression and capture each relationship through the regression coefficient. Figure 4 summarizes the results of the simple linear regression analysis using heat maps (see Figure S28 in Supporting Information S1 for an example regression plot). The most significant disproportionalities, measured through the regression coefficient, are for visible minorities and social and material deprivation. We find negative, but smaller magnitude, regression coefficients for most other vulnerable groups, with the exception of senior populations and children. LICO and South Asian are the two sub‐groups that experience the most significant disproportionality in environmental burdens and benefits. The inequity experienced by South Asian residents is also reflected in the population‐weighted means for the single environmental variables (see Tables S5 and S6 in Supporting Information S1). For both 2006 and 2016, the population‐weighted means for walkability are the lowest (0.86 compared to 3.33 in 2016) and the distance to a park or water body are the longest (449.5 m compared to 316.44 m in 2016).

For each demographic group, we also find variability in whether we identify disproportionality (negative regression coefficient) and the level of disproportionality (absolute value of the regression coefficient) across environmental combinations. Only one demographic group have negative regression coefficients for all environmental combinations—the population without certificates, diploma, or degree, as the indicator of educational level, in 2006. Some vulnerable populations, like populations over 65 years old and children from 0 to 14 years old, do not have or have limited disproportionate exposure to sour and risky spots for most combinations. However, there are also key findings for individual environmental factors; for example, we observed a lower proportion of children in sweet spots and a higher proportion in sour spots for combinations including walkability and accessibility to a park or water body, which are some of the key environmental benefits to children's health and well‐being.

At the same time, we see patterns in environmental combinations. Most groups are not disproportionately exposed to sour spots for heat stress and accessibility to a park of water body, and walkability and accessibility to a park and water body. Compared to 2006, distributional inequity for walkability and NO_2_ is alleviated for most groups. For example, for total visible minorities and material deprivation, we observed large shifts from coefficients of −11.09 and −9.15, respectively, to −3.41 and 0.86, for walkability and NO_2_, resulting from increases in the percentage of these two groups in sweet spots and decreasing percentage in risky spots.

**Figure 5:**
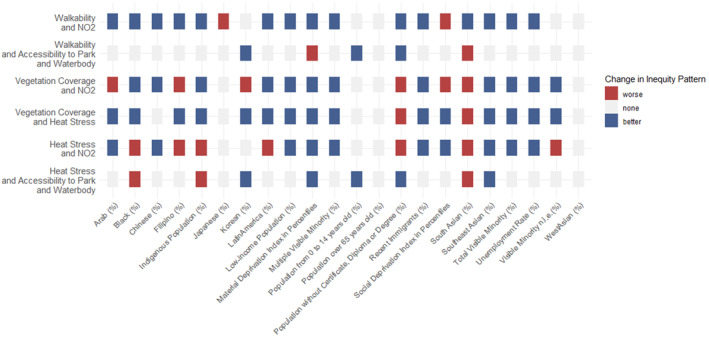
Changes in Distributional Environmental Inequity Patterns between the Years 2006 and 2016. Blue indicates decreased disproportionality for vulnerable groups, and red indicates increased disproportionality. Gray represents no changes identified from this method.

Based on the changes in regression coefficients (i.e., changes in the direction and magnitude) between the years 2006 and 2016, we identify the changing distributional patterns for each demographic group in Figure 5. Disproportionate exposure to sour and risky spots is not alleviated for all groups. For materially‐deprived populations, regression coefficients are less negative in 2016 than 2006 for 4 out of 6 environmental combinations, and change signs for walkability and NO_2_. In contrast, for socially‐deprived populations, 2 out of 6 regression coefficients are more negative in 2016 compared to 2006. Recent immigrants usually represent a higher percentage of population in sour or risky spots in 2006 (e.g., for vegetation coverage and heat stress, 7.87% in sour spots compared to 4.78% in sweet spots; for walkability and NO_2_, 7.32% in sour spots and 8.13% in risky spots compared to 2.21% in sweet spots); however, they tend to have a lower percentage in sour or risky spots and a higher percentage in sweet spots in 2016 (for vegetation coverage and heat stress, 4.93% in sour spots compared to 4.97 in sweet spots; for walkability and NO_2_, 5.26% in sour spots and 5.81% in risky spots compared to 6.93% in sweet spots). As a result, regression coefficients turn from negative to positive for 3 out of 6 environmental combinations, and the coefficient is less negative for one combination. Visible minorities experienced reduced inequities in exposure over time, but patterns vary among racialized and ethnic groups. Using Chinese and South Asian populations as examples, Chinese residents experienced alleviation (regression coefficients change from negative to positive or not statistically significantly positive) in many environmental combinations (3 out of 6), while South Asian residents faced greater inequities (more negative coefficients) in most combinations (5 out of 6).

## 4 Discussion

This study uses a two‐dimensional characterization method, sweet and sour spot analysis, to explore exposure to environmental quality across a wide range of demographic groups, and its changes between 2006 and 2016. We find that environmental quality in Metro Vancouver generally improved between 2006 and 2016; however, the environmental benefits and burdens were inequitably distributed, and this inequitable distribution did not improve for all demographic groups. Given the availability of census data, this study considers two discrete time points to assess change over time (with some consideration of interannual variability); as a result, there may be variations through the decade that are not captured in these results. Further, we note that although sweet spots and sour spots are identified as areas with favorable and unfavorable environmental conditions, respectively, these labels are relative assessments based on the local maximum and minimum environmental performance of environmental variables within this region.

For both 2006 and 2016, sweet spots for most environmental combinations were rare. This result is consistent with previous studies that focused on green space, NO_2_, and walkability in Metro Vancouver and two other major cities in Canada, one in 2016 and one in early 2000, suggesting that the rarity of sweet spots is consistent across a wide range of environmental variables and time periods (Doiron et al., 2020; Marshall et al., 2009). The number of sour spots was also generally low, but higher than sweet spots in most combinations. In addition, there are few DAs classified as sweet in terms of all environmental combinations (0.03% in 2006 and 0.18% in 2016). DAs where all combinations are sour or risky are limited as well but slightly higher (0.3% in 2006 and 0.4% in 2016). The small percentages of both sweet and sour spots suggest that there are distinct spatial patterns across the environmental variables, resulting in limited spatial overlaps for both variables at favourable or unfavourable performance. This finding highlights the importance of considering cumulative exposures in environmental assessment and management compared to single‐variable‐based ones.

In contrast with the small number of sweet spots in other combinations, we find a relatively large percentage of sweet spots in the combinations with vegetation coverage (e.g., vegetation coverage and NO_2_: 26.2%; vegetation coverage and heat stress: 21.8%, in 2006). This finding may indicate that favourable performance of vegetation coverage can reduce the adverse impacts of other variables and enhance the living experiences of residents in the environment. On the other hand, we identify a significant proportion of risky spots (around 13% in 2006 and 11% in 2016) for the walkability and NO_2_ combination, highlighting that residents in a large number of DAs are facing high level of NO_2_ exposure during their daily active transportation. Environmental policies and planning decisions can, therefore, take advantage of the interactions between environmental factors to yield cumulative health benefits or be aware of any potential trade‐offs in health outcomes.

The spatial distributions of the environmental spots changed between 2006 and 2016; we observe similar spatial patterns but identify variations in magnitude and locations. We observe different kinds of changes in each environmental combination, including newly emerged sweet or sour spots and flipping over between sour and sweet spots. Figure 2 shows the sweet and sour heatmap considering all environmental combinations; areas identified as sweet across many combinations in 2006 were sweet for more combinations in 2016, with a similar finding for sour spots. In short: the sweet spots were sweeter and the sour spots were sourer. This pattern could contribute to larger disparities in environmental exposures and could be hard to identify from commonly applied evaluation metrics like weighted mean. In urban planning literature, previous studies that focused on urban green infrastructure planning (green spaces with emphasized ecological benefits) highlight failures to meet equity‐related goals (Grabowski et al., 2023; Hoover et al., 2023) and failure to prioritize neighborhoods with the most need, potentially exacerbating existing inequity (Mahmoudi et al., 2020). There are also, however, improvements in environmental quality that are likely related to urban planning decisions and environmental‐related policies. Using walkability and NO_2_ as an example again, we find areas with high NO_2_ concentration are clustered around major roads in both years (as NO_2_ is one of the key components of TRAP); at the same time, many of these areas have high community walkability, including more roads, footpaths, and diverse facilities, potentially reducing benefits of active mobility for residents. Fortunately, NO_2_ concentration decreased in all these areas in 2016, possibly as a result of “stricter vehicle emission standards, inspection and maintenance programs” implemented by Metro Vancouver (Brauer et al., 2013; Doerksen et al., 2020).

We observe variability between groups for different environmental combinations across the 2 years. In general, material and social deprivation, total visible minorities, and South Asian residents are the groups that experience inequitable residential exposures in both years in the most combinations. A complex interplay of factors could contribute to these inequitable residential exposures. Using materially‐deprived populations and South Asian residents as examples, two groups that experienced inequitable exposure to almost all environmental combinations in both years (See Figure 4), the literature suggests that residential segregation—an outcome from multiple factors like “economic changes, institutionalized discriminatory practices in the housing market, or preferences of residents to cluster together”— could be one of the core drivers of inequitable environmental exposures, in combination with the planning and policy drivers outlined above (Kruize et al., 2014). A typical example of urban planning policy driving environmental inequity from residential segregation would be redlining policies adopted by the U.S. in the 1930s. Although it was abolished in 1968, the legacy impacts are still significant for the inequitable distribution of multiple environmental factors (Estien et al., 2024). In our study, we do find highly clustered residential spatial patterns, especially for visible minorities like Chinese and South Asians. When considering changes over time, many of the vulnerable groups that were identified as experiencing inequity in 2006 had improvement in 2016, such as materially deprived and Chinese residents, which is supported by both the ratio of percentage population in sweet to sour and the regression coefficients; however, some groups did not experience better situations, for example, South Asian residents. In addition to environmental planning decisions and policies that fail to benefit areas with vulnerable populations and unfavorable environments, another explanation from the literature is that urban planning could contribute to green gentrification, where the enhancement of environmental amenities leads to the displacement of originally less privileged and marginalized populations (Anguelovski, 2016).

In summary, the environment in Metro Vancouver is relatively favourable and it improved between 2006 and 2016. Still, distributional inequity exists in terms of these overlapping environmental burdens and benefits and deepened over time for some structurally marginalized groups. Based on these findings, this study provides several policy implications. Firstly, environmental interventions could take advantage of the favourable interactions between environmental factors; for instance, the regulating functions of urban vegetation on heat stress and air pollution mean that policies for urban green space can yield multiple benefits. However, the implementation of green space also requires careful design, to avoid any adverse impacts on local air quality due to ventilation changes (Diener & Mudu, 2021). On the other hand, NO_2_ levels should be a concern, especially for areas with high levels of walkability and other active transportation. Second, interventions should prioritize populations based on their vulnerability and susceptibility to environmental exposures and the combined effects of multiple exposures, aiming for environmental health equity (Kruize et al., 2014). For example, some groups, like populations experiencing material deprivation, are more vulnerable to the health effects of air pollution and heat stress because of their lower access to air conditioning and air filtration (Fann et al., 2016; Gamble et al., 2016; Henderson et al., 2022; Kovats & Hajat, 2008; Rahman et al., 2022). Integrating the above knowledge and our results that indicate inequity for materially‐deprived residents, we suggest prioritized intervention for heat stress and air pollution for specific “sour spots” in Metro Vancouver, taking advantage of strategies that bring together technological innovation, housing quality and security, and public health and urban planning (Yoon et al., 2024). Finally, policy and interventions should consider drivers of environmental inequity, from both environmental and social perspectives. Drivers of these changes and existing patterns are complex, and not fully captured by top‐down quantitative metrics used here, and require further interdisciplinary research. Some examples include urban planning, environmental policy, and economic factors like housing affordability (Kruize et al., 2014). We suggest that longitudinal case studies on the changing patterns of inequity will support exploring the drivers from multiple dimensions.

We identify opportunities to address limitations in this study in future research. Firstly, there are many environmental factors that show significant impacts on human physical and mental health and well‐being and are related to social injustice that are not included in this research due to lack of data availability. Additionally, there are many alternatives for the metrics that we used to operationalize environmental factors, for example, heat stress and accessibility to a park or water body, as discussed in the Methods section. Thirdly, this study uses the regression coefficients from simple linear regression as a summary metric for relative distributional inequity across groups. More regression models are available for other purposes. For example, given that the environmental category used in this study is ordinal data, ordinal logit regression is another choice to measure inequity. The modelled log odds can be interpreted as “a one‐unit increase in demographic percentage leads to a decrease/increase in the probability of a more favourable category.” However, this would yield 2 to 3 log odds from each regression given the number of categories, and the results could be challenging to summarize, visualize, and compare across a wide range of groups and environmental combinations and between years. Lastly, we specifically highlight that this regression analysis does not suggest any causal relationship; a statistically significant linear relationship does not imply that differences in environmental categories cause changes in demographic percentage, or vice versa. While the methodological framework can be applied to other cases, the identified patterns of distributional inequity are specific to Metro Vancouver for the years 2006 and 2016.

## 5 Conclusions

The characterization of environmental sweet, risky, and sour spots across different combinations highlights areas needing the most attention and reveals distributional inequities based on dual exposure to burdens and lack of benefits. In this study, we assess cumulative exposure patterns by considering the spatial distributions of two environmental factors at a time, and we identify the distinct patterns that could be missing in single‐factor exposure assessment. Building on past sweet and sour spot analyses (Doiron et al., 2020; Hankey et al., 2017), we expand the exposure assessment to five environmental factors and identify changes over a decade. This longitudinal quantitative method allows us to explore exacerbated disparities in cumulative exposures, aligning with previous findings that environmental improvements from urban planning decisions may reproduce inequity by targeting the ‘wrong’ areas (instead of those that truly need improvements) (Mahmoudi et al., 2020). While we find general improvement in environmental quality, we identify disproportionate cumulative exposures for marginalized and vulnerable groups in both 2006 and 2016, with limited or no sign of improvement for groups like the South Asian population. In conclusion, we echo calls for increasing attention to cumulative exposure to risks and access to benefits across a wide range of environmental factors in EJ research and related policy and decision‐making. Further, we argue that understanding the changing spatial patterns of environmental quality, demographic distribution, and injustice through time can contribute to the exploration of the drivers of these patterns, including historical and ongoing urban planning and policy decisions, ultimately supporting more equitable interventions.

## Conflict of Interest

The authors declare no conflicts of interest relevant to this study.

## Supporting information

Supporting Information S1

## Data Availability

The data sources of environmental and demographic variables are detailed in the Method section. The tables that integrate raw environmental and demographic data for the years 2006 and 2016 and the code to conduct all analyses in RStudio described in the Methods section are available on Zenodo Repository (Ren & Giang, 2024) (10.5281/zenodo.12670163). All the maps in this study, including Figures 1, and 2, and Figures S1, S3–S15, and S27 in Supporting Information S1, were developed using ArcGIS Pro.

## References

[gh2587-bib-0001] Agyeman, J. , Schlosberg, D. , Craven, L. , & Matthews, C. (2016). Trends and directions in environmental justice: From inequity to everyday life, community, and just sustainabilities. In Annual review of environment and resources (Vol. 41(1), pp. 321–340). Annual Reviews Inc. 10.1146/annurev-environ-110615-090052

[gh2587-bib-0002] Anguelovski, I. (2016). From toxic sites to parks as (green) LULUs? New challenges of inequity, privilege, gentrification, and exclusion for urban environmental justice. Journal of Planning Literature, 31(1), 23–36. 10.1177/0885412215610491

[gh2587-bib-0003] Brauer, M. , Reynolds, C. , & Hystad, P. (2013). Traffic‐related air pollution and health in Canada. Canadian Medical Association Journal, 185(18), 1557–1558. 10.1503/cmaj.121568 24144607 PMC3855107

[gh2587-bib-0004] Brochu, P. , Jimenez, M. P. , James, P. , Kinney, P. L. , & Lane, K. (2022). Benefits of increasing greenness on all‐cause mortality in the largest metropolitan areas of the United States within the past two decades. Frontiers in Public Health, 10, 1250. 10.3389/fpubh.2022.841936 PMC9127575 35619828

[gh2587-bib-0005] Brulle, R. J. , & Pellow, D. N. (2006). Environmental justice: Human health and environmental inequalities. Annual Review of Public Health, 27(1), 103–124. 10.1146/annurev.publhealth.27.021405.102124 16533111

[gh2587-bib-0006] Brusseau, M. L. , Ramirez‐Andreotta, M. , Pepper, I. L. , & Maximillian, J. (2019). Environmental impacts on human health and well‐being. In Environmental and pollution science (pp. 477–499). Elsevier. 10.1016/b978-0-12-814719-1.00026-4

[gh2587-bib-0007] Campbell, C. (2020). There’s something in the water: Environmental racism in indigenous and Black communities. Journal of the Royal Nova Scotia Historical Society, 23, 126–128. https://www.proquest.com/scholarly‐journals/theres‐something‐water‐environmental‐racism/docview/2550550113/se‐2?accountid=14656

[gh2587-bib-0008] Canadian Census Analyser at CHASS . (2021). 2006 and 2016 census profile of census dissemination areas cansim (database) using chass (distributor).

[gh2587-bib-0010] Clark, L. P. , Millet, D. B. , & Marshall, J. D. (2017). Changes in transportation‐related air pollution exposures by race‐ethnicity and socioeconomic status: Outdoor nitrogen dioxide in the United States in 2000 and 2010. Environmental Health Perspectives, 125(9). 10.1289/EHP959 PMC5915204 28930515

[gh2587-bib-0011] Copernicus Climate Change Service (C3S) . (2017). ERA5: Fifth generation of ECMWF atmospheric reanalyses of the global climate. Copernicus Climate Change Service Climate Data Store (CDS).

[gh2587-bib-0012] Craig, K. (2021). Social equity and regional growth study: Considerations for integrating social equity into regional planning and Metro 2050. Retrieved from https://metrovancouver.org/services/regional‐planning/Documents/metro‐vancouver‐social‐equity‐regional‐growth‐study.pdf

[gh2587-bib-0013] Crouse, D. L. , Pinault, L. , Balram, A. , Brauer, M. , Burnett, R. T. , Martin, R. V. , et al. (2019). Complex relationships between greenness, air pollution, and mortality in a population‐based Canadian cohort. Environment International, 128, 292–300. 10.1016/j.envint.2019.04.047 31075749

[gh2587-bib-0014] Davis, R. E. , McGregor, G. R. , & Enfield, K. B. (2016). Humidity: A review and primer on atmospheric moisture and human health. In Environmental research (Vol. 144, pp. 106–116). Academic Press Inc. 10.1016/j.envres.2015.10.014 26599589

[gh2587-bib-0015] Davis, Z. , de Groh, M. , & Rainham, D. G. (2022). The Canadian Environmental Quality Index (Can‐EQI): Development and calculation of an index to assess spatial variation of environmental quality in Canada’s 30 largest cities. Environment International, 170, 107633. 10.1016/j.envint.2022.107633 36413927

[gh2587-bib-0016] Diener, A. , & Mudu, P. (2021). How can vegetation protect us from air pollution? A critical review on green spaces’ mitigation abilities for air‐borne particles from a public health perspective ‐ With implications for urban planning. In Science of the total environment (Vol. 796, 148605), Elsevier B.V. 10.1016/j.scitotenv.2021.148605 34271387

[gh2587-bib-0017] DMTI Spatial Inc . (2010). Canmap parks, v2009.3. In Abacus data Network (V1 ed.). D. S. Inc.

[gh2587-bib-0018] DMTI Spatial Inc . (2014). CanMap parks, v2014.3. In Abacus data Network (V1 ed.). D. S. Inc.

[gh2587-bib-0019] DMTI Spatial Inc . (2015). CanMap RouteLogistics, v2014.3. Abacus data Network. https://hdl.handle.net/11272.1/AB2/HQIQEB

[gh2587-bib-0020] Doerksen, G. , Howe, K. , Thai, A. , & Reid, K. (2020). Metro vancouver near‐road air quality monitoring study. https://metrovancouver.org/services/air‐quality‐climate‐action/Documents/near‐road‐air‐quality‐monitoring‐study‐technical‐report‐2015‐2017.pdf

[gh2587-bib-0021] Doiron, D. , Setton, E. M. , Shairsingh, K. , Brauer, M. , Hystad, P. , Ross, N. A. , & Brook, J. R. (2020). Healthy built environment: Spatial patterns and relationships of multiple exposures and deprivation in Toronto, Montreal and Vancouver. Environment International, 143, 106003. 10.1016/j.envint.2020.106003 32763633

[gh2587-bib-0022] Environment and Climate Change Canada . (2017a). Air quality research division, Toronto, Canada. Data files: GEMMACH_GroundLevel_O3_NA_2010.nc to GEMMACH_Ground‐Level_O3_NA_2015.nc inclusive.

[gh2587-bib-0023] Environment and Climate Change Canada . (2017b). Air quality research division, Toronto, Canada. Data files: CHRONOS_GroundLevel_O3_NA_2002.nc to CHRONOS_Ground‐Level_O3_NA_2009.nc inclusive.

[gh2587-bib-0024] Environment and Climate Change Canada . (2017c). Air quality research division, Toronto, Canada. Data files: OMI_GroundLevel_SO2_NA_2005.nc to OMI_Ground‐Level_SO2_NA_2015.nc inclusive.

[gh2587-bib-0025] Environment and Climate Change Canada . (2023). Update – Strengthening the Canadian Environmental Protection Act, 1999 and recognizing a right to a healthy environment.

[gh2587-bib-0026] Estien, C. O. , Wilkinson, C. E. , Morello‐Frosch, R. , & Schell, C. J. (2024). Historical redlining is associated with disparities in environmental quality across California. Environmental Science and Technology Letters, 11(2), 54–59. 10.1021/acs.estlett.3c00870 38371654 PMC10867848

[gh2587-bib-0027] Fann, N. , Brennan, T. , Dolwick, P. , Gamble, J. L. , Ilacqua, V. , Kolb, L. , et al. (2016). Ch. 3: Air quality impacts. In The impacts of climate change on human health in the United States: A scientific assessment. 10.7930/J0GQ6VP6

[gh2587-bib-0028] Franchini, M. , & Mannucci, P. M. (2018). Mitigation of air pollution by greenness: A narrative review. In European journal of internal medicine (Vol. 55, pp. 1–5). Elsevier B.V. 10.1016/j.ejim.2018.06.021 30180945

[gh2587-bib-0029] Frank, L. D. , Chair, B. , Devlin, A. , Johnstone, S. , & Van Loon, J. (2010). Neighbourhood design, travel, and health in Metro vancouver: Using a walkability index. Retrieved from https://atl.sites.olt.ubc.ca/files/2011/06/WalkReport_ExecSum_Oct2010_HighRes.pdf

[gh2587-bib-0030] Gamache, P. , Hamel, D. , & Blaser, C. (2019). Material and social deprivation index: A summary – INSPQ website. www.inspq.qc.ca/en/publications/2639

[gh2587-bib-0031] Gamble, J. L. , Balbus, J. , Berger, M. , Bouye, K. , Campbell, V. , Chief, K. , et al. (2016). Ch. 9: Populations of concern. In The impacts of climate change on human health in the United States: A scientific assessment (pp. 247–286). U.S. Global Change Research Program. 10.7930/J0Q81B0T

[gh2587-bib-0032] Gary, V. , Gray, B. , Yanchapaxi, F. , Bos, K. , & Murphy, M. (2023). Data colonialism in Canada’s chemical valley: Aamjiwnaang first nation and the failure of the pollution notification system. https://yellowheadinstitute.org/wp‐content/uploads/2023/09/Data‐Colonialism‐YI‐Special‐Report‐Sept‐2023‐3‐compressed‐1.pdf

[gh2587-bib-0033] Gascon, M. , Mas, M. T. , Martínez, D. , Dadvand, P. , Forns, J. , Plasència, A. , & Nieuwenhuijsen, M. J. (2015). Mental health benefits of long‐term exposure to residential green and blue spaces: A systematic review. In International journal of environmental research and public health (Vol. 12, pp. 4354–4379). MDPI. 10.3390/ijerph120404354 25913182 PMC4410252

[gh2587-bib-0104] Gasparrini, A. , & Armstrong, B. (2011). The impact of heat waves on mortality. Epidemiology, 22(1), 68–73. 10.1097/EDE.0b013e3181fdcd99 21150355 PMC3324776

[gh2587-bib-0035] Giang, A. , Boyd, D. R. , Ono, A. J. , & McIlroy‐Young, B. (2022). Exposure, access, and inequities: Central themes, emerging trends, and key gaps in Canadian environmental justice literature from 2006 to 2017. Canadian Geographer, 66(3), 434–449. 10.1111/cag.12754

[gh2587-bib-0036] Giang, A. , & Castellani, K. (2020). Cumulative air pollution indicators highlight unique patterns of injustice in urban Canada. Environmental Research Letters, 15(12), 124063. 10.1088/1748-9326/abcac5

[gh2587-bib-0037] Google Earth Engine Explorer . (2017a). Landsat 5 TM annual greenest‐pixel TOA reflectance composite, 1984 to 2012. Retrieved from https://explorer.earthengine.google.com/detail/LANDSAT/LT5_L1T_ANNUAL_GREENEST_TOA

[gh2587-bib-0038] Google Earth Engine Explorer . (2017b). Landsat 8 annual greenest‐pixel TOA reflectance composite, 2013 to 2015. Retrieved from https://explorer.earthengine.google.com/detail/LANDSAT/LC8_L1T_ANNUAL_GREENEST_TOA

[gh2587-bib-0039] Gorelick, N. , Hancher, M. , Dixon, M. , Ilyushchenko, S. , Thau, D. , & Moore, R. (2017). Google Earth Engine: Planetary‐scale geospatial analysis for everyone. Remote Sensing of Environment.

[gh2587-bib-0040] Grabowski, Z. J. , McPhearson, T. , & Pickett, S. T. A. (2023). Transforming US urban green infrastructure planning to address equity. Landscape and Urban Planning, 229, 104591. 10.1016/j.landurbplan.2022.104591

[gh2587-bib-0041] Grow, H. M. , Saelens, B. E. , Kerr, J. , Durant, N. H. , Norman, G. J. , & Sallis, J. F. (2008). Where are youth active? Roles of proximity, active transport, and built environment. Medicine & Science in Sports & Exercise, 40(12), 2071–2079. 10.1249/MSS.0b013e3181817baa 18981942

[gh2587-bib-0042] Guo, Y. , Chen, Z. , Stuart, A. , Li, X. , & Zhang, Y. (2020). A systematic overview of transportation equity in terms of accessibility, traffic emissions, and safety outcomes: From conventional to emerging technologies. In Transportation research interdisciplinary perspectives (Vol. 4). 100091, Elsevier Ltd. 10.1016/j.trip.2020.100091

[gh2587-bib-0043] Hammer, M. S. , Van Donkelaar, A. , Li, C. , Lyapustin, A. , Sayer, A. M. , Hsu, N. C. , et al. (2020). Global estimates and long‐term trends of fine particulate matter concentrations (1998‐2018). Environmental Science and Technology, 54(13), 7879–7890. 10.1021/acs.est.0c01764 32491847

[gh2587-bib-0044] Hankey, S. , Lindsey, G. , & Marshall, J. D. (2017). Population‐level exposure to particulate air pollution during active travel: Planning for low‐exposure, health‐promoting cities. Environmental Health Perspectives, 125(4), 527–534. 10.1289/EHP442 27713109 PMC5381994

[gh2587-bib-0045] Hankey, S. , & Marshall, J. D. (2017). Urban form, air pollution, and health. In Current environmental health reports (Vol. 4(4), 491–503). Springer. 10.1007/s40572-017-0167-7 29052114

[gh2587-bib-0046] Hankey, S. , Marshall, J. D. , & Brauer, M. (2012). Health impacts of the built environment: Within‐urban variability in physical inactivity, air pollution, and ischemic heart disease mortality. Environmental Health Perspectives, 120(2), 247–253. 10.1289/ehp.1103806 22004949 PMC3279444

[gh2587-bib-0047] Henderson, S. B. , McLean, K. E. , Lee, M. J. , & Kosatsky, T. (2022). Analysis of community deaths during the catastrophic 2021 heat dome. Environmental Epidemiology, 6(1), E189. 10.1097/EE9.0000000000000189 35169667 PMC8835552

[gh2587-bib-0048] Ho, H. C. , Knudby, A. , Xu, Y. , Hodul, M. , & Aminipouri, M. (2016). A comparison of urban heat islands mapped using skin temperature, air temperature, and apparent temperature (Humidex), for the greater Vancouver area. Science of the Total Environment, 544, 929–938. 10.1016/j.scitotenv.2015.12.021 26706765

[gh2587-bib-0049] Hoffmann, B. , Boogaard, H. , de Nazelle, A. , Andersen, Z. J. , Abramson, M. , Brauer, M. , et al. (2021). WHO air quality guidelines 2021–aiming for healthier air for all: A joint statement by medical, public health, scientific societies and patient representative organisations. International Journal of Public Health, 66. 10.3389/ijph.2021.1604465 PMC8494774 34630006

[gh2587-bib-0050] Hoover, F. A. , Meerow, S. , Coleman, E. , Grabowski, Z. , & McPhearson, T. (2023). Why go green? Comparing rationales and planning criteria for green infrastructure in U.S. City plans. Landscape and Urban Planning, 237, 104781. 10.1016/j.landurbplan.2023.104781

[gh2587-bib-0051] Howell, N. A. , Tu, J. V. , Moineddin, R. , Chen, H. , Chu, A. , Hystad, P. , & Booth, G. L. (2019). Interaction between neighborhood walkability and traffic‐related air pollution on hypertension and diabetes: The CANHEART cohort. Environment International, 132, 104799. 10.1016/j.envint.2019.04.070 31253484

[gh2587-bib-0052] Hystad, P. , Setton, E. , Cervantes, A. , Poplawski, K. , Deschenes, S. , Brauer, M. , et al. (2011). Creating national air pollution models for population exposure assessment in Canada. Environmental Health Perspectives, 119(8), 1123–1129. 10.1289/ehp.1002976 21454147 PMC3237350

[gh2587-bib-0054] James, P. , Kioumourtzoglou, M. A. , Hart, J. E. , Banay, R. F. , Kloog, I. , & Laden, F. (2017). Interrelationships between walkability, air pollution, greenness, and body mass index. Epidemiology, 28(6), 780–788. 10.1097/EDE.0000000000000724 28767514 PMC5617802

[gh2587-bib-0055] Kovats, R. S. , & Hajat, S. (2008). Heat stress and public health: A critical review. Annual Review of Public Health, 29(1), 41–55. 10.1146/annurev.publhealth.29.020907.090843 18031221

[gh2587-bib-0056] Kruize, H. , Droomers, M. , van Kamp, I. , & Ruijsbroek, A. (2014). What causes environmental inequalities and related health effects? An analysis of evolving concepts. In International journal of environmental research and public health (Vol. 11(6), 5807–5827). MDPI. 10.3390/ijerph110605807 24886752 PMC4078549

[gh2587-bib-0057] Lafortezza, R. , Carrus, G. , Sanesi, G. , & Davies, C. (2009). Benefits and well‐being perceived by people visiting green spaces in periods of heat stress. Urban Forestry and Urban Greening, 8(2), 97–108. 10.1016/j.ufug.2009.02.003

[gh2587-bib-0058] Lane, H. M. , Morello‐Frosch, R. , Marshall, J. D. , & Apte, J. S. (2022). Historical redlining is associated with present‐day air pollution disparities in U.S. Cities. Environmental Science and Technology Letters, 9(4), 345–350. 10.1021/acs.estlett.1c01012 35434171 PMC9009174

[gh2587-bib-0059] Maguire, K. , & Sheriff, G. (2011). Comparing distributions of environmental outcomes for regulatory environmental justice analysis. International Journal of Environmental Research and Public Health, 8(5), 1707–1726. 10.3390/ijerph8051707 21655146 PMC3108136

[gh2587-bib-0060] Mahmoudi, D. , Lubitow, A. , & Christensen, M. A. (2020). Reproducing spatial inequality? The sustainability fix and barriers to urban mobility in portland, Oregon. Urban Geography, 41(6), 801–822. 10.1080/02723638.2019.1698865

[gh2587-bib-0061] Maroko, A. R. , Maantay, J. A. , Sohler, N. L. , Grady, K. L. , & Arno, P. S. (2009). The complexities of measuring access to parks and physical activity sites in New York city: A quantitative and qualitative approach. International Journal of Health Geographics, 8(1), 34. 10.1186/1476-072X-8-34 19545430 PMC2708147

[gh2587-bib-0062] Marshall, J. D. , Brauer, M. , & Frank, L. D. (2009). Healthy neighborhoods: Walkability and air pollution. Environmental Health Perspectives, 117(11), 1752–1759. 10.1289/ehp.0900595.S1 20049128 PMC2801167

[gh2587-bib-0063] McLinden, C. A. , Fioletov, V. , Boersma, K. F. , Kharol, S. K. , Krotkov, N. , Lamsal, L. , et al. (2014). Improved satellite retrievals of NO2 and SO2 over the Canadian oil sands and comparisons with surface measurements. Atmospheric Chemistry and Physics, 14(7), 3637–3656. 10.5194/acp-14-3637-2014

[gh2587-bib-0064] Metro Vancouver . (2011). Metro Vancouver 2040 shaping our future: Regional growth strategy, Bylaw No.1136, 2010. Metro Vancouver. Retrieved from https://metrovancouver.org/about‐us/Documents/regional‐growth‐strategy‐metro‐2040.pdf#search=Regional%20growth%20strategy

[gh2587-bib-0065] Metro Vancouver . (2021a). Regional park boundary. Retrieved from https://arcg.is/19DyCW

[gh2587-bib-0066] Metro Vancouver . (2021b). Regional parks history timeline (1966 to present). Retrieved from https://web.archive.org/web/20221205185003/http://www.metrovancouver.org/services/parks/stories/timeline/Pages/default.aspx

[gh2587-bib-0067] Mohai, P. , & Saha, R. (2015). Which came first, people or pollution? A review of theory and evidence from longitudinal environmental justice studies. Environmental Research Letters, 10(12), 125011. 10.1088/1748-9326/10/12/125011

[gh2587-bib-0068] Morello‐Frosch, R. , Zuk, M. , Jerrett, M. , Shamasunder, B. , & Kyle, A. D. (2011). Understanding the cumulative impacts of inequalities in environmental health: Implications for policy. Health Affairs, 30(5), 879–887. 10.1377/hlthaff.2011.0153 21555471

[gh2587-bib-0069] Mueller, N. , Rojas‐Rueda, D. , Cole‐Hunter, T. , de Nazelle, A. , Dons, E. , Gerike, R. , et al. (2015). Health impact assessment of active transportation: A systematic review. In Preventive medicine (Vol. 76, pp. 103–114). Academic Press Inc. 10.1016/j.ypmed.2015.04.010 25900805

[gh2587-bib-0070] Nguyen, P. Y. , Astell‐Burt, T. , Rahimi‐Ardabili, H. , & Feng, X. (2021). Green space quality and health: A systematic review. In International journal of environmental research and public health (Vol. 18(21), 11028). MDPI. 10.3390/ijerph182111028 34769549 PMC8582763

[gh2587-bib-0071] Nieuwenhuijsen, M. J. (2016). Urban and transport planning, environmental exposures and health‐new concepts, methods and tools to improve health in cities. In Environmental health, environmental health: A global access science source (Vol. 15(S1), S38). BioMed Central Ltd. 10.1186/s12940-016-0108-1 PMC4895603 26960529

[gh2587-bib-0072] Pan‐Canadian Public Health Network . (2018). Key health inequalities in Canada: A national portrait. Public Health Agency of Canada = Agence de santé publique du Canada.

[gh2587-bib-0073] Park, K. , Rigolon, A. , Choi, D. , Lyons, T. , & Brewer, S. (2021). Transit to parks: An environmental justice study of transit access to large parks in the U.S. West. Urban Forestry and Urban Greening, 60, 127055. 10.1016/j.ufug.2021.127055

[gh2587-bib-0074] Pinault, L. , Crouse, D. , Jerrett, M. , Brauer, M. , & Tjepkema, M. (2016). Spatial associations between socioeconomic groups and NO2 air pollution exposure within three large Canadian cities. Environmental Research, 147(2), 373–382. 10.1016/j.envres.2016.02.033 26950027

[gh2587-bib-0075] Racz, L. A. , & Rish, W. (2022). Exposure monitoring toward environmental justice. Integrated Environmental Assessment and Management, 18(4), 858–862. 10.1002/ieam.4534 34633140

[gh2587-bib-0076] Rahman, M. M. , McConnell, R. , Schlaerth, H. , Ko, J. , Silva, S. , Lurmann, F. W. , et al. (2022). The effects of Co‐exposure to extremes of heat and particulate air pollution on mortality in California implications for climate change. American Journal of Respiratory and Critical Care Medicine, 206(9), 1117–1127. 10.1164/rccm.202204-0657OC 35727303 PMC9704834

[gh2587-bib-0077] Ren, S. , & Giang, A. (2024). Supplemental data for Inequitable spatial and temporal patterns in the distribution of multiple environmental risks and benefits in Metro Vancouver [Dataset]. Zenodo. 10.5281/zenodo.12670163 PMC11659194 39712528

[gh2587-bib-0078] Richardson, A. S. , Ghosh‐Dastidar, M. , Collins, R. L. , Hunter, G. P. , Troxel, W. M. , Colabianchi, N. , et al. (2020). Improved street walkability, incivilities, and esthetics are associated with greater park use in two low‐income neighborhoods. Journal of Urban Health, 97(2), 204–212. 10.1007/s11524-019-00416-7 31989419 PMC7101449

[gh2587-bib-0079] Robichaud, A. , & Ménard, R. (2014). Multi‐year objective analyses of warm season ground‐level ozone and PM2.5 over North America using real‐time observations and Canadian operational air quality models. Atmospheric Chemistry and Physics, 14(4), 1769–1800. 10.5194/acp-14-1769-2014

[gh2587-bib-0080] Robichaud, A. , Ménard, R. , Zaïtseva, Y. , & Anselmo, D. (2016). Multi‐pollutant surface objective analyses and mapping of air quality health index over North America. Air Quality, Atmosphere and Health, 9(7), 743–759. 10.1007/s11869-015-0385-9 PMC5054062 27785157

[gh2587-bib-0081] Ross, N. , Wasfi, R. , Herrmann, T. , & Gleckner, W. (2018). Canadian active living environments database (Can‐ALE) user manual.

[gh2587-bib-0082] Sax, D. L. , Nesbitt, L. , & Hagerman, S. (2022). Expelled from the garden? Understanding the dynamics of green gentrification in vancouver, British Columbia. Environment and Planning: Nature and Space, 6(3), 2008–2028. 10.1177/25148486221123134

[gh2587-bib-0083] Silva, M. A. D. , Gravel, N. , Sylvain‐Morneau, J. , Blaser, C. , Gamache, P. , & Hamel, D. (2024). Material and social deprivation index 2021 USER MANUAL. Retrieved from https://www.inspq.qc.ca/sites/default/files/2024‐04/3476‐material‐social‐deprivation‐index‐guide‐2021.pdf

[gh2587-bib-0084] Statistics Canada . (2022). Canada’s large urban centres continue to grow and spread.

[gh2587-bib-0085] Statistics Canada . (2023). Census profile. 2021 census of population. Statistics Canada catalogue no. 98‐316‐X2021001. Ottawa. Retrieved from https://www12.statcan.gc.ca/census‐recensement/2021/dp‐pd/prof/index.cfm?Lang=E

[gh2587-bib-0086] Stieb, D. M. , Smith‐Doiron, M. , Quick, M. , Christidis, T. , Xi, G. , Miles, R. M. , et al. (2023). Inequality in the distribution of air pollution attributable mortality within Canadian cities. GeoHealth, 7(9). 10.1029/2023GH000816 PMC10465848 37654974

[gh2587-bib-0087] Stossel, Z. , Kissinger, M. , & Meir, A. (2015). Assessing the state of environmental quality in cities ‐ a multi‐component urban performance (EMCUP) index. Environmental Pollution, 206, 679–687. 10.1016/j.envpol.2015.07.036 26334706

[gh2587-bib-0088] Su, J. G. , Morello‐Frosch, R. , Jesdale, B. M. , Kyle, A. D. , Shamasunder, B. , & Jerrett, M. (2009). An index for assessing demographic inequalities in cumulative environmental hazards with application to Los Angeles, California. Environmental Science and Technology, 43(20), 7626–7634. 10.1021/es901041p 19921871

[gh2587-bib-0089] The World Bank . (2023). Urban population (% of total population). United Nations population division. In World urbanization prospects: 2018 revision. License : CC BY‐4.0.

[gh2587-bib-0090] Thornton, M. M. , Shrestha, R. , Wei, Y. , Thornton, P. E. , Kao, S.‐C. , & Wilson, B. E. (2022). Daymet: Monthly climate summaries on a 1‐km grid for North America, *version 4* .

[gh2587-bib-0103] UN. General Assembly (76th sess. : 2021‐2022) . (2022). The human right to a clean, healthy and sustainable environment : Resolution / adopted by the General Assembly. Retrieved from https://digitallibrary.un.org/record/3983329?ln=en#record‐files‐collapse‐header

[gh2587-bib-0091] USGS . (2017a). USGS landsat 5 TM TOA reflectance (orthorectified), 1984 to 2011. Retrieved from https://explorer.earthengine.google.com/detail/LANDSAT/LT5_L1T_TOA

[gh2587-bib-0092] USGS . (2017b). USGS landsat 8 TOA reflectance (orthorectified), 2013 to 2017. Retrieved from https://explorer.earthengine.google.com/detail/LANDSAT/LC8_L1T_TOA

[gh2587-bib-0093] Waldron, I. (2022). Environmental racism and climate change: Determinants of health in mi’kmaw and african nova scotian communities. Retrieved from https://climateinstitute.ca/publications/environmental‐racism‐and‐climate‐change/

[gh2587-bib-0094] Walker, G. (2010). Environmental justice, impact assessment and the politics of knowledge: The implications of assessing the social distribution of environmental outcomes. Environmental Impact Assessment Review, 30(5), 312–318. 10.1016/j.eiar.2010.04.005

[gh2587-bib-0095] Weichenthal, S. , Pinault, L. L. , & Burnett, R. T. (2017). Impact of oxidant gases on the relationship between outdoor fine particulate air pollution and nonaccidental, cardiovascular, and respiratory mortality. Scientific Reports, 7(1), 16401. 10.1038/s41598-017-16770-y 29180643 PMC5703979

[gh2587-bib-0096] White, M. P. , Elliott, L. R. , Gascon, M. , Roberts, B. , & Fleming, L. E. (2020). Blue space, health and well‐being: A narrative overview and synthesis of potential benefits. In Environmental research (Vol. 191). 110169, Academic Press Inc. 10.1016/j.envres.2020.110169 32971082

[gh2587-bib-0097] World Health Organization . (2021). WHO global air quality guidelines: Particulate matter (PM2.5 and PM10), ozone, nitrogen dioxide, sulfur dioxide and carbon monoxide. World Health Organization. Retrieved from https://apps.who.int/iris/handle/10665/345329 34662007

[gh2587-bib-0098] Yao, J. , Stieb, D. M. , Taylor, E. , & Henderson, S. B. (2020). Assessment of the air quality health index (AQHI) and four alternate AQHI‐plus amendments for wildfire seasons in British Columbia. Canadian Journal of Public Health, 111(1), 96–106. 10.17269/s41997-019-00237-w 31286460 PMC7046905

[gh2587-bib-0099] Yoon, L. , Sun, L. , Kulka, R. , White, K. , Stern, R. , Chakraborty, M. , et al. (2024). Assessing indoor environmental quality and energy insecurity in low‐income housing: An inter‐disciplinary and citizen science approach. In International Society of Exposure Science (ISES) 2024 Annual Conference.

[gh2587-bib-0100] Zhang, K. , Li, Y. , Schwartz, J. D. , & O’Neill, M. S. (2014). What weather variables are important in predicting heat‐related mortality? A new application of statistical learning methods. Environmental Research, 132, 350–359. 10.1016/j.envres.2014.04.004 24834832 PMC4091921

[gh2587-bib-0101] Zupancic, T. , Westmacott, C. , & Bulthuis, M. (2015). The impact of green space on heat and air pollution in urban communities: A meta‐narrative systematic review.

