# Supplementary material for: Inequitable Spatial and Temporal Patterns in the Distribution of Multiple Environmental Risks and Benefits in Metro Vancouver
Source: Geohealth. 2024 Dec 19;8(12):e2024GH001157. doi: 10.1029/2024GH001157 (PMC11659194; doi:10.1029/2024GH001157)
Supplement: Supplementary file 1 — Supporting Information S1 [file GH2-8-e2024GH001157-s001.pdf]

GeoHealth

Supporting Information for

Spatial and temporal patterns in the distribution of multiple urban environmental risks and benefits in Metro Vancouver

Shuoqi Ren[1], Amanda Giang[1,2]

[1]Institute for Resources, Environment and Sustainability, University of British Columbia, Vancouver, Canada

[2]Department of Mechanical Engineering, University of British Columbia, Vancouver, Canada

Contents of this file

Text S1 to S4
Figures S1 to S32
Tables S1 to S9

Introduction

This Supporting Information document provides 1) Text S1 - the definition of urban areas and Figure S1 - the maps of the study area for 2006 and 2016, 2) Text S2 - the description of demographic variables; 3) Text S3 - the differences between the quartile method and even break method, 4) Figure S2 - an illustrative figure for the classification of sweet, risky, and sour spots using the combination of walkability and NO_2. 5) Table S1 to S4 - the descriptive statistics of environmental and demographic variables for 2006 and 2016, 6) Tables S5 and S6 - The population-weighted average of environmental variables for different demographic groups in the years 2006 and 2016. 7) Table S7 - ranges of absolute values of environmental variables in each combination, 8) Table S8 – definition of environmental distributional inequity patterns from the results of simple linear regression between environmental spots category and demographic statistics, 9) Table S9 – table showing the proportions of sweet, sour, risky, and medium spots for each environmental combination. 10) Figure S3 to S10 – maps for the spatial distribution of 8 environmental variables for Metro Vancouver in the year 2006. 11) Figure S11 to S15 – maps showing sweet and sour spot spatial distribution for all environmental combinations, 12) Figure S17 to S26 - descriptive statistics of demographic variables (including social deprivation, material deprivation, Indigenous population, total visible minorities, white population, population without higher education, unemployment rate, recent immigrants, low-income population, population over 65 years old, and population

from 0-14 years old) across environment spots with violin and box plots, 13) Figure S27 - sweet and sour heat maps for 2006 and 2016 from even breaks method. 14) Figure 28 – an example regression plot of vegetation coverage and NO_2 vs low-income population percentage for the year 2016. 15) Figure S29 and S30 – the results of distributional inequity identification from the even breaks methods. 16) Figure S31 and S32 - the results of distributional inequity identification from the regression analysis without risky spots (as a sensitivity test) and Text S4 – discussion about the robustness and differences based on the sensitivity test.

Text S1. Definition of urban areas in this study

In this study, we only analyzed urban areas in Metro Vancouver. The definitions of urban and rural areas in this study are from Statistics Canada (2023). Urban areas are population centers, which are areas with "least 1,000 in total population and a population density of 400 persons or more per square kilometre." Rural areas are "all areas outside population centres." We linked and aggregated the population centers with the DA based on the Dissemination Geographies Relationship File for the 2021 census year (Statistics Canada, 2021). Rural areas include ocean, forests, and cropland with large areas and low population density. Around 0.02% of DA (count for around 60% of total areas) defined as rural areas in Metro Vancouver and eliminated from the analysis.

Text S2. Description of demographic variables

The detailed metrics we used for demographic variables for 2006 and 2016 are from Canadian Census data, accessed through the Canadian Census analyzer (Canadian Census Analyser at CHASS, 2021), and INSPQ (Gamache et al., 2019; Institut national de santé publique du Québec (INSPQ)., n.d.) are listed below. All the variables are at dissemination area (DA) resolution.

Age: We include children and older people as biologically susceptible populations by including the population's broad age groups of 0 to 14 years recognized as children and 65 years and over recognized as older people, based on 100% census data for both sexes.

Low-income status: We recognize the low-income status of a DA from the prevalence of low income based on the Low-income cut-offs (after tax), i.e. LICO, which is the most commonly used metric to represent low-income status in Canada. The definition of a low-income family under LICO is "the family that will devote a larger share (more than 20%) of its income on the necessities of food, shelter and clothing than the average family" (Canada. Employment and Social Development Canada., 2016).

Recent Immigrant: The population of recent immigrants is based on 25% sample data for 2016 and 20% sample data for 2006 on recent 5-year immigration, i.e. immigrants from 2011 to 2016 for the 2016 Census and 2001 to 2006 for the 2006 Census.

Race/ ethnicity: We include white, total visible minority, and Indigenous identity (Aboriginal identity) as the three main categories for ethnicity. The populations of the total visible minority and Indigenous identity are based on 25% of sample data for 2016 and 20% of sample data for 2006, and the population of White is the population other than visible minorities and Indigenous identify. The total visible minority further includes 12 ethnic/racialized groups, which are South Asian, Chinese, Black, Filipino, Latin American, Arab, Southeast Asian, West Asian, Korean, Japanese, visible minorities not included elsewhere (visible minority n.i.e), and multiple visible minorities. The populations of the 12 ethnic groups are based on 100% census data.

Educational attainment: We derived the population that does not hold any certificate, diploma or degree and is aged 15 years and over based on the 25% of sample data for 2016 and 20% of sample data for 2006.

Employment Status: We include the unemployment rate for each DA.

Deprivation Index: We used two composite deprivation indices from INSPQ (Gamache et al., 2019; Institut national de santé publique du Québec (INSPQ)., n.d.), one representing social deprivation and the other one representing material deprivation. Social deprivation refers to "a fragile social network, starting with the family and encompassing the community. It is characterized by "individuals living alone, being a lone parent and being separated, divorced or widowed" (Gamache et al., 2019). Material deprivation refers to the "deprivation of the goods and conveniences that are part of modern life, such as adequate housing, possession of a car, access to high-speed internet, or a neighbourhood with recreational areas" (Gamache et al., 2019). The other three variables we include, which are low-income status, unemployment rate, and educational attainment, are associated with material deprivation. The Indices we derived are based on the percentile in the national range of Canada, ranging from 1 as the least deprived DA and 100 as the most deprived DA (Gamache et al., 2019).

Text S3. Differences between the quartile method and even break method

To categorize the environment of each DA into sweet, sour, risky, and medium, we applied two methods, even breaks and quartile, to classify DAs into four categories for each environmental variable. These two methods lead to different results determined by the data structure. In the even breaks method, the variables with high skewness and kurtosis and extreme outliers result in very few numbers of DA in the best or worst category, making identification of statistically significant relationships between demographics and environmental category more challenging, and leading to bias in injustice identification (for example, walkability and NO_2). As a result, we introduce

results from the quartile method in the main manuscript and include the results from the even breaks method in this SI. However, the even breaks method can highlight how residents in some areas are facing extremely unfavourable environments compared to the general public. According to the data structure and purpose, future studies in other cities or focused on other variables could apply other methods. The spatial distributions of sweet, sour, and risky spots identified from the even breaks method are slightly different from the ones identified from the quartile method, and this difference leads to the discrepancy in inequity identification. Except for the results that include variables with extremely high skewness and kurtosis (e.g. NO_2 and walkability), the differences are limited. As an example, the heat map of sweet and sour spots from the even break method (SI Figure S27) illustrates that a smaller number of DA are recognized as being "sweet" or "sour" at the same time for multiple environmental combinations. The reason could be extreme values in environmental factors leading to few DAs identified as sweet or sour from the even break method. The spatial patterns are visually similar, with more sweet spots in the City of Vancouver and West Vancouver, and more sour spots in Surrey and Langley.

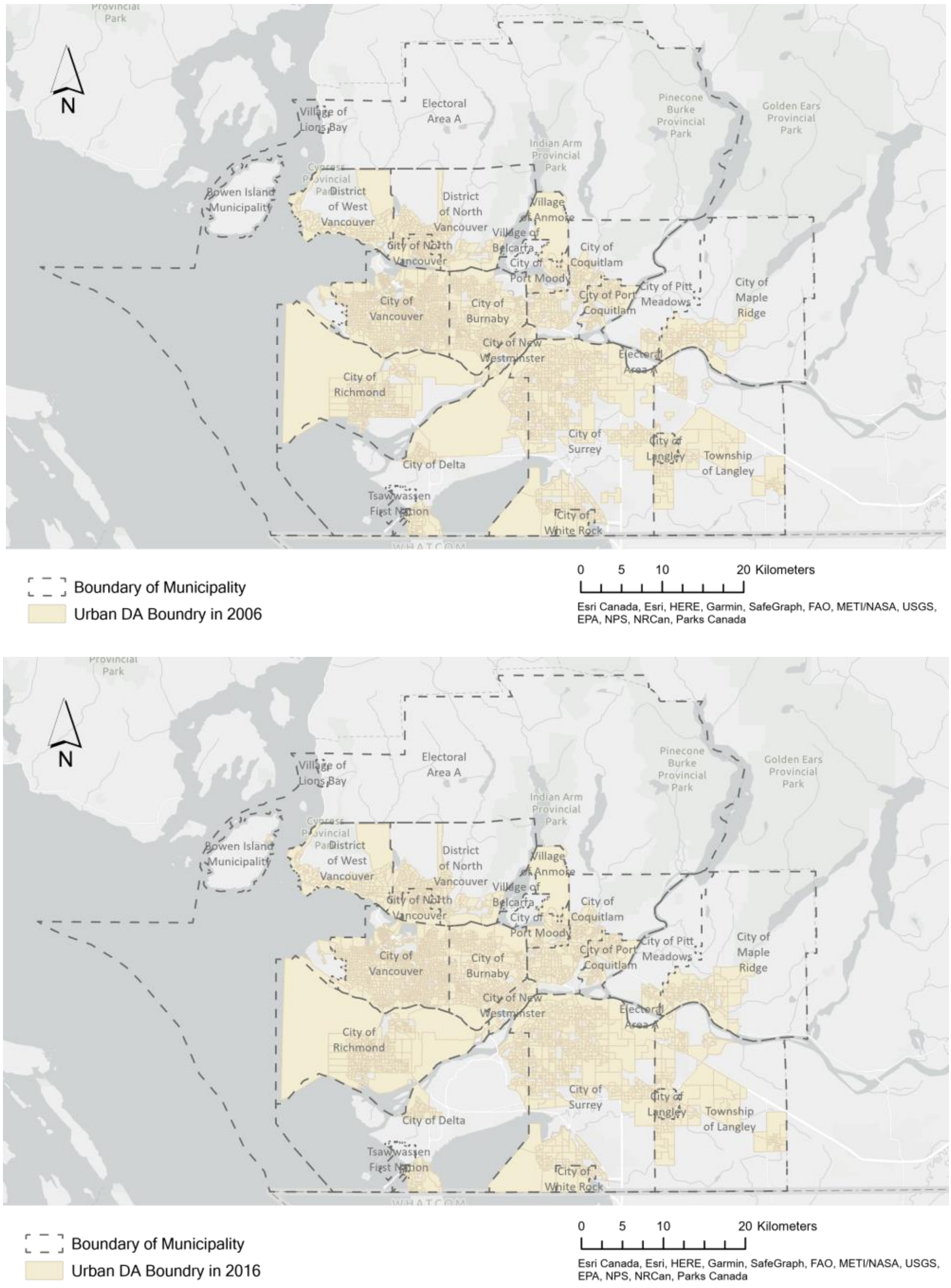

Figure S1. Map for the studied urban area in Metro Vancouver for 2006 and 2016

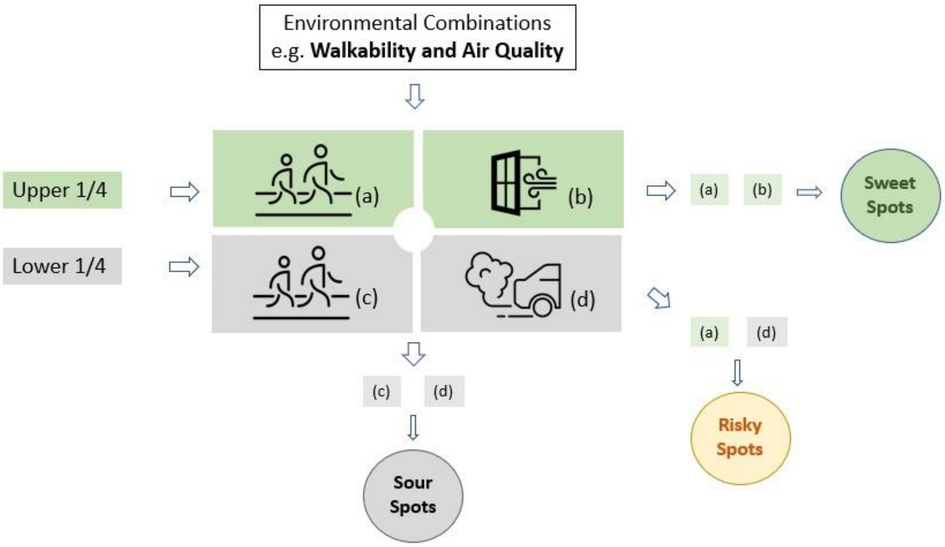

Figure S2. An Illustrative figure for the classification of sweet, risky, and sour spots using the combination of walkability and NO_2.

Table S1. Descriptive Statistics of Environmental Variables in 2006. All the values are rounded to two decimal points. The P-value is lower than 0.001 shown as *.**

The Year 2006	Minimum	Maximum	Population-weighted Mean	Median	First Quartile	Third Quartile	CV	Skewness	Kurtosis	Moran's I
NO2 Concentration (ppb)	6.21	37.51	16.84	17.04	13.29	19.93	0.28	0.19	3.06	0.93***
PM 2.5 Concentration (ug/m3)	5.74	8.17	7.29	7.27	7.05	7.47	0.05	-0.01	3.75	0.95***
Ozone Concentration (ppb)	23.39	33.68	25.13	24.87	23.39	25.35	0.09	1.76	5.94	0.95***
SO2 Concentration (ppb)	0.14	0.73	0.25	0.22	0.21	0.30	0.27	1.90	13.71	0.88***
Apparent Temperature (Celsius degree)	25.32	28.58	27.24	27.21	27.01	27.42	0.02	0.36	4.62	0.93***
Vegetation Coverage (NDVI)	0.04	0.64	0.33	0.34	0.29	0.39	0.25	-0.50	3.79	0.63***
Walkability	-2.03	10.27	1.16	0.80	0.05	2.15	1.53	1.50	6.66	0.92***
Accessibility to a Park or Water Body (m)	0.00	2268.02	370.09	256.86	156.88	427.56	0.85	2.11	8.62	0.74***

Table S2. Descriptive Statistics of Environmental Variables in 2016. All the values are rounded to two decimal points. The P-value is lower than 0.001 shown as ***.

The Year 2016	Minimum	Maximum	Population-weighted Mean	Median	First Quartile	Third Quartile	CV	Skewness	Kurtosis	Moran's I
NO2 Concentration (ppb)	4.00	31.33	13.02	13.04	10.20	15.21	0.28	0.21	3.20	0.95***
PM 2.5 Concentration (ug/m3)	4.04	7.11	5.8	5.80	5.53	6.09	0.08	-0.25	3.62	0.92***
Ozone Concentration (ppb)	22.09	38.14	25.5	25.71	22.73	27.24	0.11	0.72	3.48	0.89***
SO2 Concentration (ppb)	0.29	0.85	0.48	0.46	0.41	0.50	0.23	1.49	5.35	0.93***
Apparent Temperature (Celsius degree)	24.01	27.43	26.4	26.35	26.16	26.62	0.02	-0.39	5.01	0.60***
Vegetation Coverage (NDVI)	0.03	0.64	0.3	0.32	0.27	0.38	0.27	-0.27	3.53	0.95***
Walkability	-2.12	25.09	1.92	0.72	-0.14	2.20	2.07	3.23	15.88	0.77***
Accessibility to a Park or Water Body (m)	0.00	2198.50	391.53	256.72	155.43	434.07	0.87	2.12	8.56	0.91***

Table S3. Descriptive Statistics of Demographic Variables in 2006. Mean, CV, skewness, and kurtosis are rounded to two decimal points.

The Year 2006	Minimum	Maximum	Mean	Median	CV	Skewness	Kurtosis
**Population from 0 to 14 years old**	0	1250	103.92	90	0.69	4.13	38.90
**Population over 65 years old**	0	775	81.19	65	0.87	3.56	21.56
**Recent Immigrants**	0	760	45.33	25	1.36	3.72	25.58
**Unemployment Rate (%)**	0	33.3	5.40	4.9	0.78	1.01	5.16
**Population without Certificate, Diploma or Degree (%)**	0	410	63.72	55	0.72	1.95	10.11
**Indigenous Population**	0	835	11.55	0	2.31	14.33	359.21
**Total Visible Minority**	0	2625	264.30	210	0.89	2.39	14.43
**Chinese**	0	2035	115.50	60	1.27	2.75	18.54
**South Asian**	0	1910	62.69	15	2.12	5.44	49.57
**Black**	0	170	6.06	0	2.28	4.52	33.77
**Filipino**	0	625	23.63	10	1.68	3.85	31.91
**Latin America**	0	165	6.66	0	2.09	3.38	19.40
**Southeast Asian**	0	250	9.93	0	2.12	3.67	23.33
**Arab**	0	130	2.16	0	4.10	6.61	61.39
**West Asian**	0	675	8.20	0	3.14	9.48	173.99
**Korean**	0	415	13.12	0	2.53	5.48	45.43
**Japanese**	0	170	7.33	0	1.88	3.55	23.32
**Visible Minority n.i.e.**	0	100	0.83	0	4.88	9.06	145.84
**Multiple Visible Minority**	0	155	6.42	0	2.05	3.66	23.31
**White**	0	4425	350.08	320	0.70	3.43	35.60
**LICO**	0	85.3	15.78	13.9	0.74	1.18	5.49
**Material Deprivation Index in Percentiles**	0	100	43.56	42	0.67	0.18	1.77
**Social Deprivation Index in Percentiles**	0	100	45.54	41	0.67	0.26	1.81

Table S4. Descriptive Statistics of Demographic Variables in 2016. All the values are rounded to two decimal points.

The Year 2016	Minimum	Maximum	Mean	Median	CV	Skewness	Kurtosis
Population from 0 to 14 years old	0	1495	105.52	85	0.91	5.72	55.83
Population over 65 years old	0	1015	112.18	90	0.79	3.50	22.12
Recent Immigrants	0	1060	42.11	30	1.32	5.11	55.18
Unemployment Rate (%)	0	33.3	5.89	5.6	0.59	0.96	6.99
Population without Certificate, Diploma or Degree (%)	0	885	83.96	65	0.79	2.75	19.19
Indigenous Population	0	960	17.79	10	1.75	12.71	310.02
Total Visible Minority	0	5010	350.86	265	1.01	4.19	34.94
Chinese	0	3115	140.70	80	1.40	4.60	40.60
South Asian	0	3495	86.01	30	2.14	6.92	88.00
Black	0	270	8.81	0	1.82	4.29	39.12
Filipino	0	630	36.71	15	1.49	3.71	25.88
Latin America	0	150	10.19	0	1.58	2.70	13.86
Southeast Asian	0	265	13.27	0	1.72	3.27	19.67
Arab	0	345	4.80	0	3.18	9.66	158.43
West Asian	0	935	13.45	0	2.74	9.58	166.22
Korean	0	840	15.52	0	2.71	8.50	114.58
Japanese	0	220	8.86	0	1.52	3.51	32.62
Visible Minority n.i.e.	0	65	1.85	0	3.07	4.36	28.74
Multiple Visible Minority	0	230	10.43	10	1.47	3.29	25.92
White	0	4950	339.43	290	0.86	4.23	39.26
LICO	0	85.5	13.57	12.3	0.60	1.76	9.90
Material Deprivation Index in Percentiles	1	100	46.12	45	0.63	0.08	1.74
Social Deprivation Index in Percentiles	1	100	44.70	39	0.67	0.37	1.86

Table S5. The Population-weighted Average of Environmental Variables for Different Demographic Groups in 2006. The highest and lowest values are highlighted in bold.

Population-weighted Mean for 2006	NO2 Concentration (ppb)	PM 2.5 Concentration (ug/m3)	Ozone Concentration (ppb)	SO2 Concentration (ppb)	Apparent Temperature (Celsius degree)	Green space (NDVI)	Walkability	Accessibility to recreational areas (m)
**Population from 0 to 14 years old**	16.59	7.30	25.20	0.25	27.26	**0.34**	0.89	375.65
**Population over 65 years old**	16.82	7.28	25.24	0.25	27.19	0.33	1.21	335.28
**Recent Immigrants**	17.92	7.22	24.70	0.26	27.20	0.31	1.49	340.75
**Unemployment Rate**	17.65	7.26	24.94	0.25	27.23	0.32	1.35	342.33
**Population without Certificate, Diploma or Degree**	17.44	7.30	25.13	0.25	27.29	0.33	1.07	368.30
**Indigenous Population**	18.02	7.34	25.18	0.24	**27.33**	0.31	1.34	343.45
**Total Visible Minority**	18.20	7.22	24.62	0.26	27.22	0.32	1.32	347.58
**Chinese**	18.21	7.16	**24.31**	**0.27**	27.19	0.32	1.48	319.79
**South Asian**	18.45	**7.36**	25.09	0.24	27.26	0.33	**0.74**	**420.00**
**Black**	18.27	7.30	25.10	0.25	27.30	0.31	1.53	335.98
**Filipino**	18.71	7.23	24.66	0.26	27.28	0.31	1.57	315.57
**Latin America**	18.53	7.23	24.74	0.25	27.25	0.30	1.78	312.04
**Southeast Asian**	**19.57**	7.21	24.38	0.26	27.32	0.31	1.50	350.71
**Arab**	18.37	7.26	24.68	0.26	27.25	0.30	1.60	310.76
**West Asian**	16.31	**7.09**	24.41	**0.24**	**27.07**	0.32	1.34	313.30
**Korean**	16.68	7.21	24.94	0.26	27.24	0.32	1.19	407.85
**Japanese**	16.97	7.21	24.95	0.25	27.13	0.33	**1.88**	**297.11**
**Visible Minority n.i.e.**	18.75	7.23	24.76	0.25	27.21	**0.30**	1.46	320.31
**Multiple Visible Minority**	18.21	7.22	24.66	0.26	27.23	0.32	1.43	330.35
**White**	**15.93**	7.32	**25.46**	0.24	27.23	0.34	1.12	356.05
**LICO**	18.12	7.23	24.69	0.25	27.22	0.31	1.67	327.00

Table S6. The Population-weighted Average of Environmental Variables for Different Demographic Groups in 2016. The highest and lowest values are highlighted in bold.

Population-weighted Mean for 2016	NO2 Concentration (ppb)	PM 2.5 Concentration (ug/m3)	Ozone Concentration (ppb)	SO2 Concentration (ppb)	Apparent Temperature (Celsius degree)	Green space (NDVI)	Walkability	Accessibility to recreational areas (m)
Population from 0 to 14 years old	12.66	5.82	25.72	0.48	26.41	**0.32**	1.20	404.17
Population over 65 years old	12.70	5.79	25.50	0.48	26.37	0.31	1.66	356.47
Recent Immigrants	13.51	5.81	25.06	0.47	26.34	0.30	2.49	368.39
Unemployment Rate	13.15	5.82	25.35	0.48	26.38	0.31	1.88	370.45
Population without Certificate, Diploma or Degree	13.30	5.88	25.51	0.48	26.44	0.31	1.36	389.30
Indigenous Population	13.02	**5.93**	**25.79**	0.48	**26.50**	0.31	1.69	371.52
Total Visible Minority	13.70	5.82	25.06	0.48	26.37	0.30	1.77	379.98
Chinese	13.54	5.75	**24.71**	**0.50**	26.31	0.32	1.80	355.86
South Asian	13.92	5.91	25.65	0.46	26.48	0.29	**0.86**	**449.50**
Black	13.80	5.92	25.40	0.46	26.46	0.30	2.19	372.48
Filipino	14.20	5.90	24.90	0.48	26.43	0.30	1.97	354.80
Latin America	13.95	5.85	24.92	0.46	26.39	0.30	3.02	330.69
Southeast Asian	**14.39**	5.91	24.93	0.47	26.42	0.30	1.96	376.45
Arab	13.64	5.90	25.38	0.46	26.41	**0.28**	**3.33**	377.96
West Asian	12.65	**5.71**	25.43	**0.44**	**26.16**	0.30	2.90	**316.44**
Korean	13.05	5.82	25.27	0.47	26.35	0.30	2.47	432.23
Japanese	13.33	5.75	25.00	0.47	26.33	0.31	2.92	317.01
Visible Minority n.i.e.	13.70	5.88	25.20	0.46	26.41	0.30	2.40	344.99
Multiple Visible Minority	13.85	5.83	24.92	0.48	26.36	0.31	2.06	368.14
White	**12.26**	5.80	25.78	0.48	26.40	0.32	1.93	367.75
LICO	13.62	5.81	24.98	0.48	26.35	0.30	2.65	350.36

Table S7. The ranges of environmental variables in sweet and sour spots for 6 environmental combinations (results from quartile method).

	Ranges of environmental variables	Sour spots		Risky spots		Sweet spots		Medium spots	
		2006	2016	2006	2016	2006	2016	2006	2016
**Walkability & NO_2**	NO_2 concentration (ppb)	20.04~30.96	15.24~22.11	19.93~37.51	15.22~31.33	10.54~12.25	6.63~10.00	6.21~34.72	4.00~26.75
	Walkability	-2.0~0.03	-2.11 ~ -0.17	2.14~9.27	2.19~25.09	2.16~2.72	2.19~4.86	-2.03~10.27	-2.12~22.88
**Heat stress & NO_2**	NO_2 concentration (ppb)	17.04~37.51	13.04~31.33	-	-	6.21~13.29	4.00~10.18	6.32~33.08	4.83~26.31
	Humidex (℃)	27.21~28.03	26.35~27.16	-	-	25.32~27.01	24.01~26.16	25.76~28.58	24.85~27.43
**Vegetation Coverage & NO_2**	NO_2 concentration (ppb)	19.94~37.51	15.23~31.33	-	-	6.21~17.02	4.00~13.00	6.96~33.40	4.83~26.31
	NDVI	0.04~0.29	0.03~0.27	-	-	0.34~0.64	0.32~0.64	0.09~0.58	0.03~0.62
**Vegetation Coverage & heat stress**	NDVI	0.01~0.29	0.08~0.27	-	-	0.34~0.58	0.32~0.64	0.04~0.64	0.03~0.62
	Humidex (℃)	27.42~28.41	26.62~27.43	-	-	25.32~27.21	24.01~26.35	25.96~28.58	24.48~27.42
**Walkability & accessibility to park and waterbody**	Walkability	-2.03~0.79	-2.12~0.70	-	-	0.80~10.27	0.71~25.09	-2.03~9.52	-2.11~23.67
	Euclidean distance (m)	257.59~2268.02	258.89~2198.50	-	-	10.28~257.20	9.11~256.61	0.00~1289.47	0.00~1571.84
**Heat stress & accessibility to park and waterbody**	Humidex (℃)	27.21~28.58	26.35~27.43	-	-	25.32~27.01	24.01~26.16	25.67~28.54	24.28~27.42
	Euclidean distance (m)	257.59~2231.72	258.76~2198.50	-	-	22.02~157.23	0.00~155.76	0.00~2268.02	0.00~1968.20

Table S8. Definition of Environmental Distributional Inequity Patterns from the Results of Simple Linear Regression Between Each Environmental Spots Category and Demographic Statistics.

		Environmental Inequity Pattern		
		**Inequity Identified**	**Not Significant**	**Not Identified**
**Linear Relationship with Environmental Quality**	**Vulnerable Population/Demographics**	Negative linear relationship	p-value > 0.05	Positive linear relationship
	**Other Population**	Positive linear relationship	p-value > 0.05	Negative linear relationship

Table S9 The statistics for sweet, sour, and risky spots for all environmental combinations (results from the quartile method)

	Sour Spots			Risky Spots			Sweet Spots			Medium Spots		
	2006 (% of total DA)	2016 (% of total DA)	change in %	2006 (% of total DA)	2016 (% of total DA)	change in %	2006 (% of total DA)	2016 (% of total DA)	change in %	2006 (% of total DA)	2016 (% of total DA)	change in %
**Walkability & NO_2**	3.31	2.79	-0.52	12.77	11.15	-1.63	0.00	0.54	0.54	83.92	85.52	1.61
**Heat Stress & NO_2**	21.93	14.74	-7.19	-	-	-	12.50	7.82	-4.68	65.57	77.44	11.86
**Vegetation Coverage & NO_2**	12.31	9.60	-2.71	-	-	-	26.23	22.80	-3.43	61.46	67.60	6.14
**Vegetation Coverage & Heat Stress**	5.20	6.48	1.28	-	-	-	21.78	21.22	-0.55	73.03	72.29	-0.73
**Walkability & Accessbility to Park and Waterbody**	24.22	22.98	-1.24	-	-	-	23.82	22.71	-1.11	51.96	54.31	2.35
**Heat Stress & Accessbility to Park and Waterbody**	22.46	24.44	1.98	-	-	-	7.86	8.59	0.73	69.69	66.97	-2.71

Note: The total count of DA included as the urban area was 3233 in 2006 and 3364 in 2016

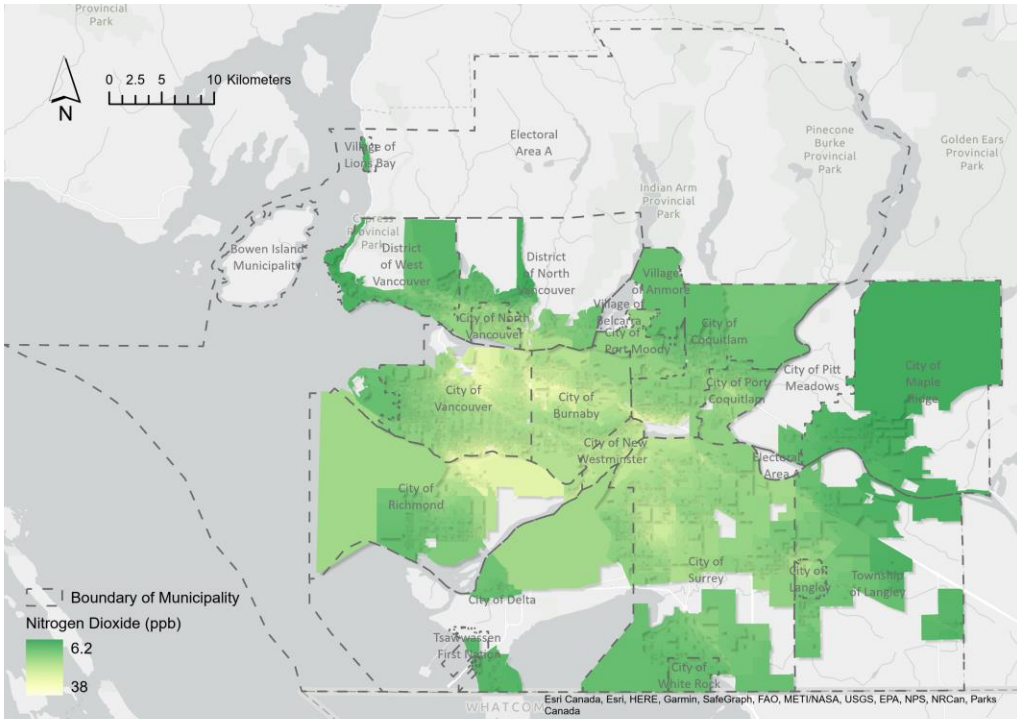

Figure S3. Spatial Distribution of NO_2 in Metro Vancouver for 2006

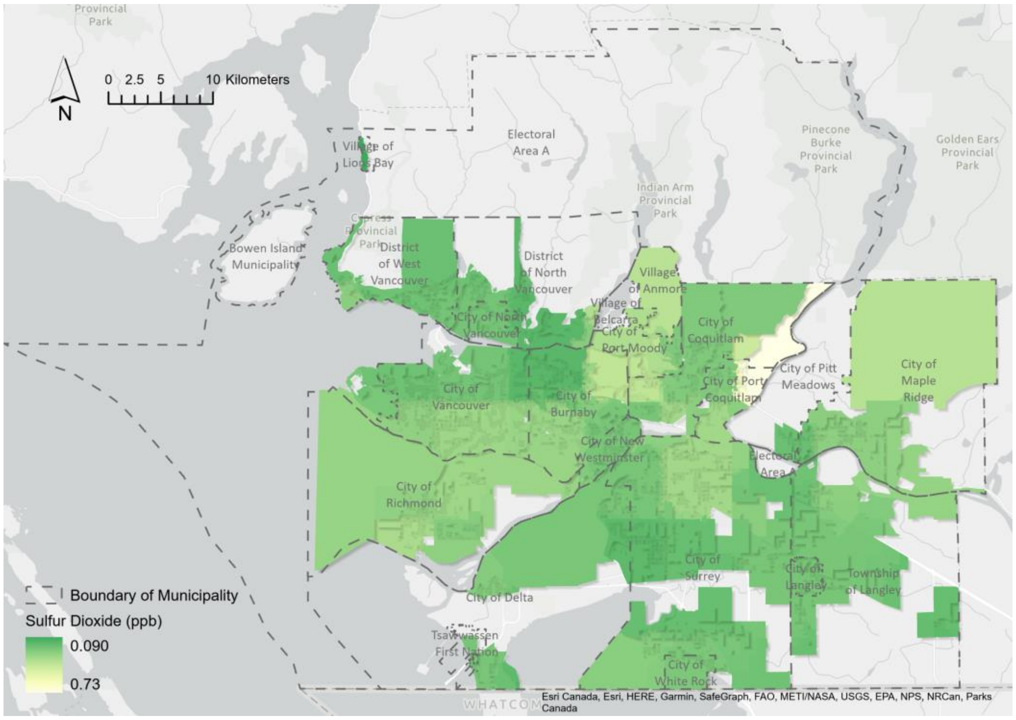

Figure S4. Spatial Distribution of SO_2 in Metro Vancouver for 2006

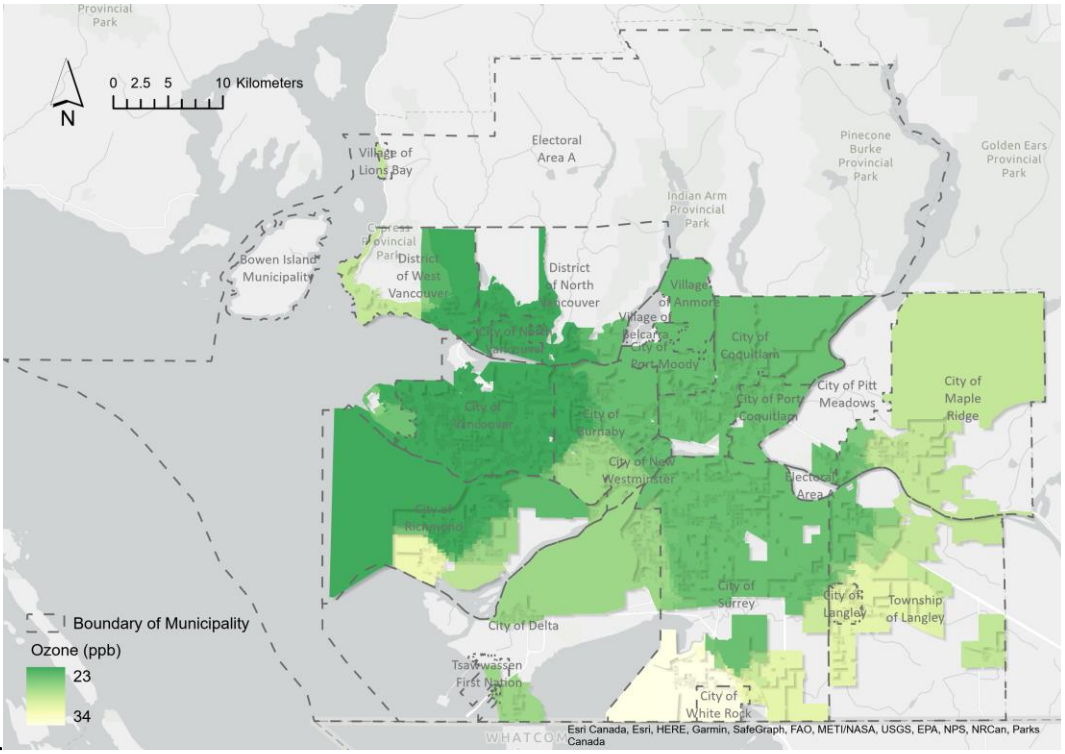

Figure S5. Spatial Distribution of O_3 in Metro Vancouver for 2006

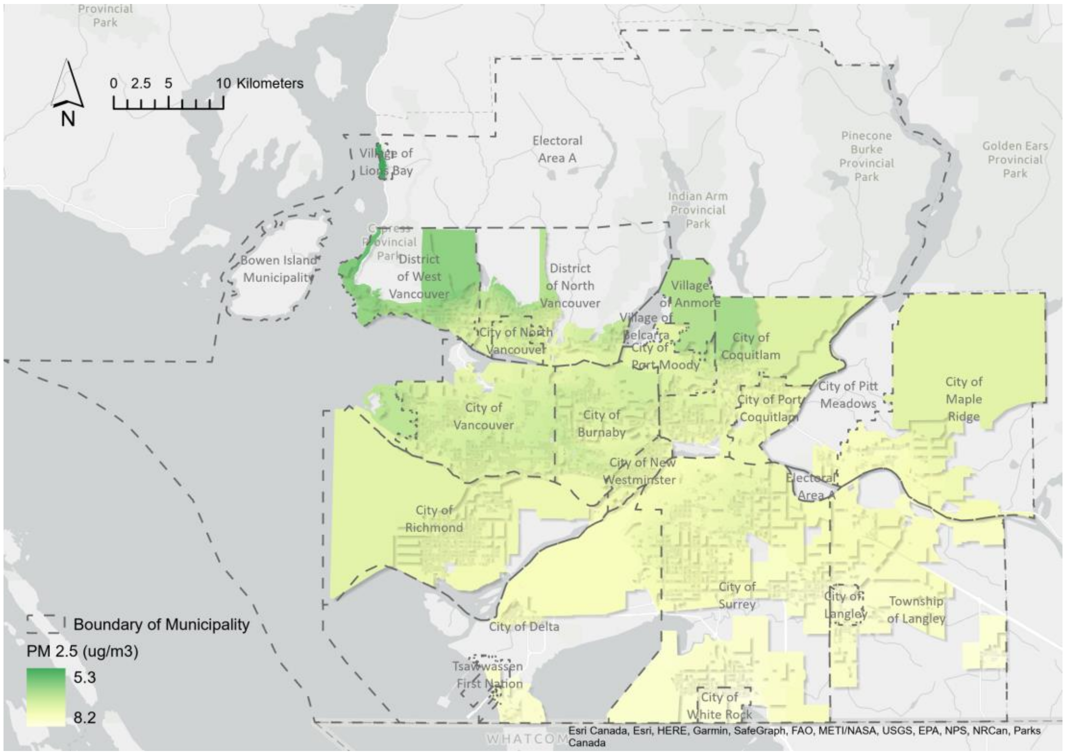

Figure S6. Spatial Distribution of $PM_{2.5}$ in Metro Vancouver for 2006

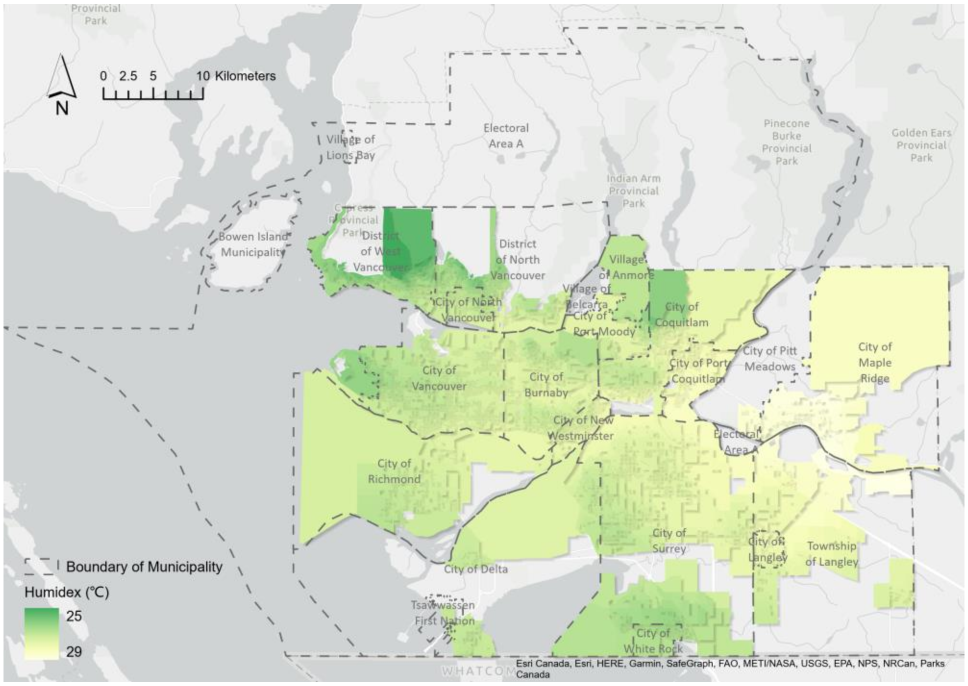

Figure S7. Spatial Distribution of Apparent Temperature/Humidex in Metro Vancouver for 2006

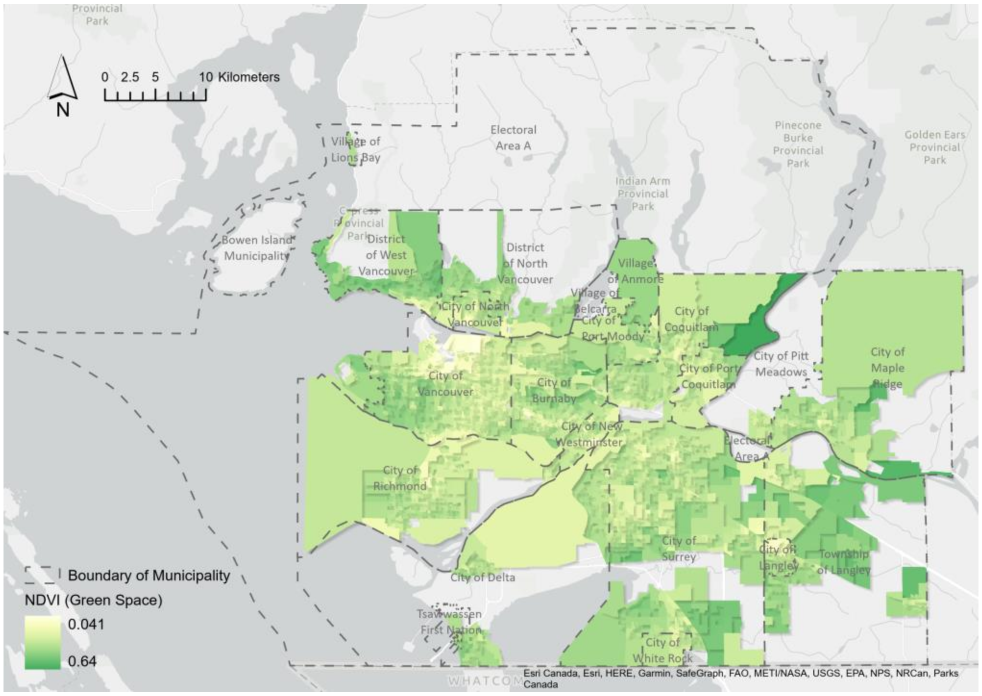

Figure S8. Spatial Distribution of Vegetation Coverage (NDVI) in Metro Vancouver for 2006

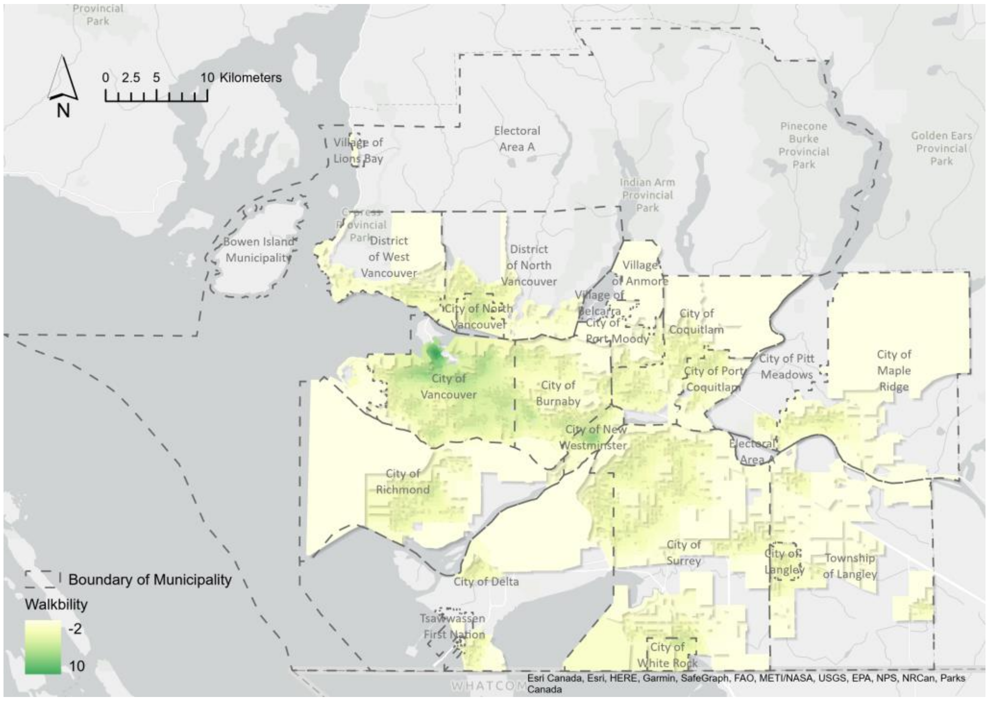

Figure S9. Spatial Distribution of Walkability in Metro Vancouver for 2006

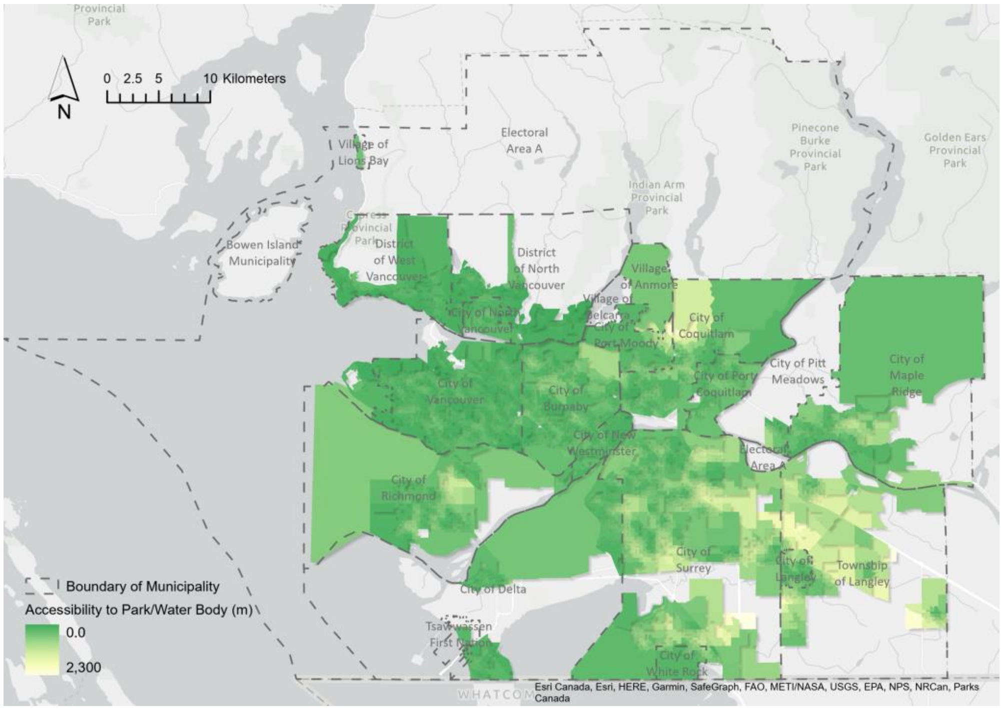

Figure S10. Spatial Distribution of Accessibility to a park or water body in Metro Vancouver for 2006

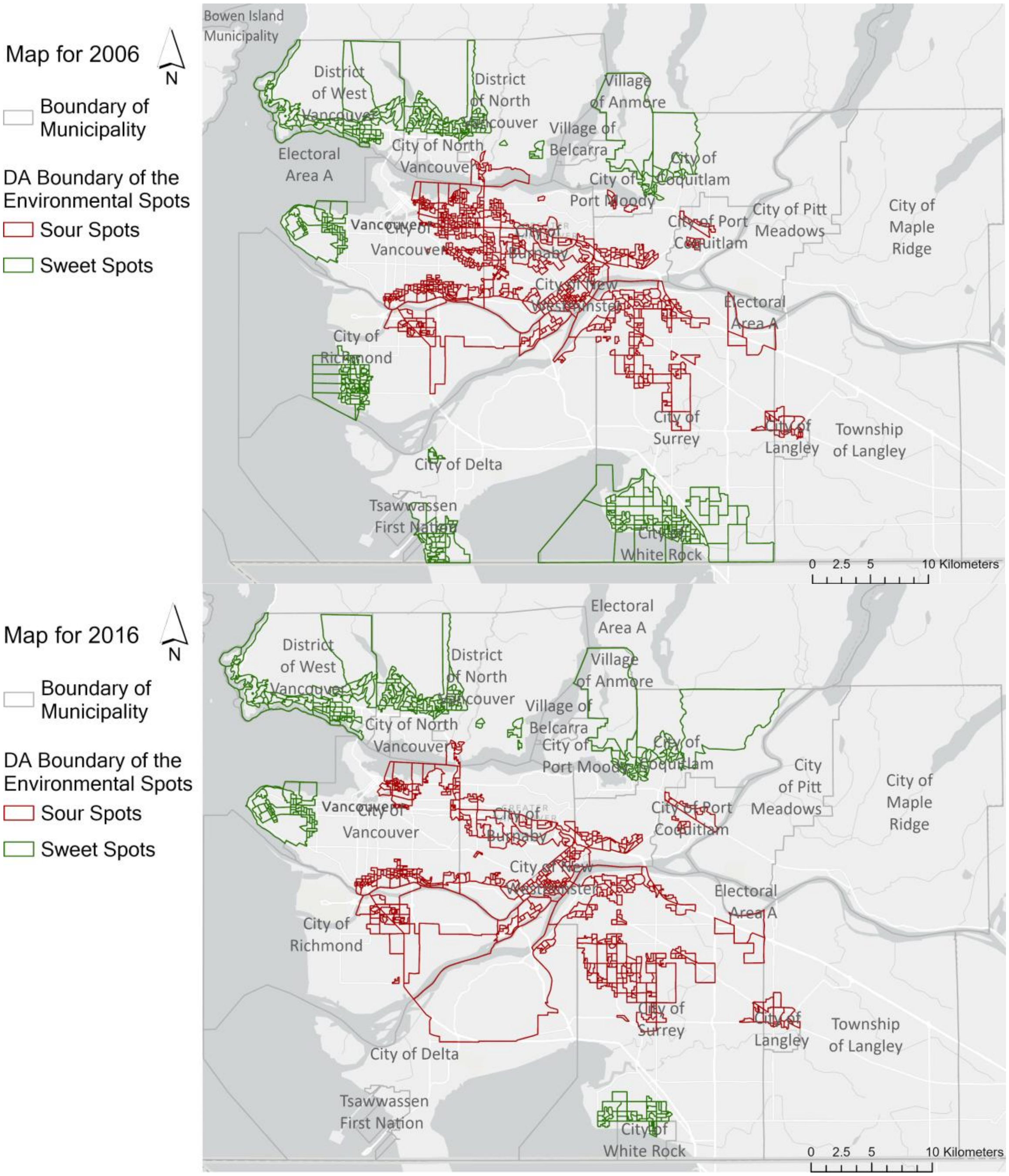

Figure S11. Spatial Distribution of Sweet and Sour Spots for Heat Stress & NO_2 in 2006 and 2016.

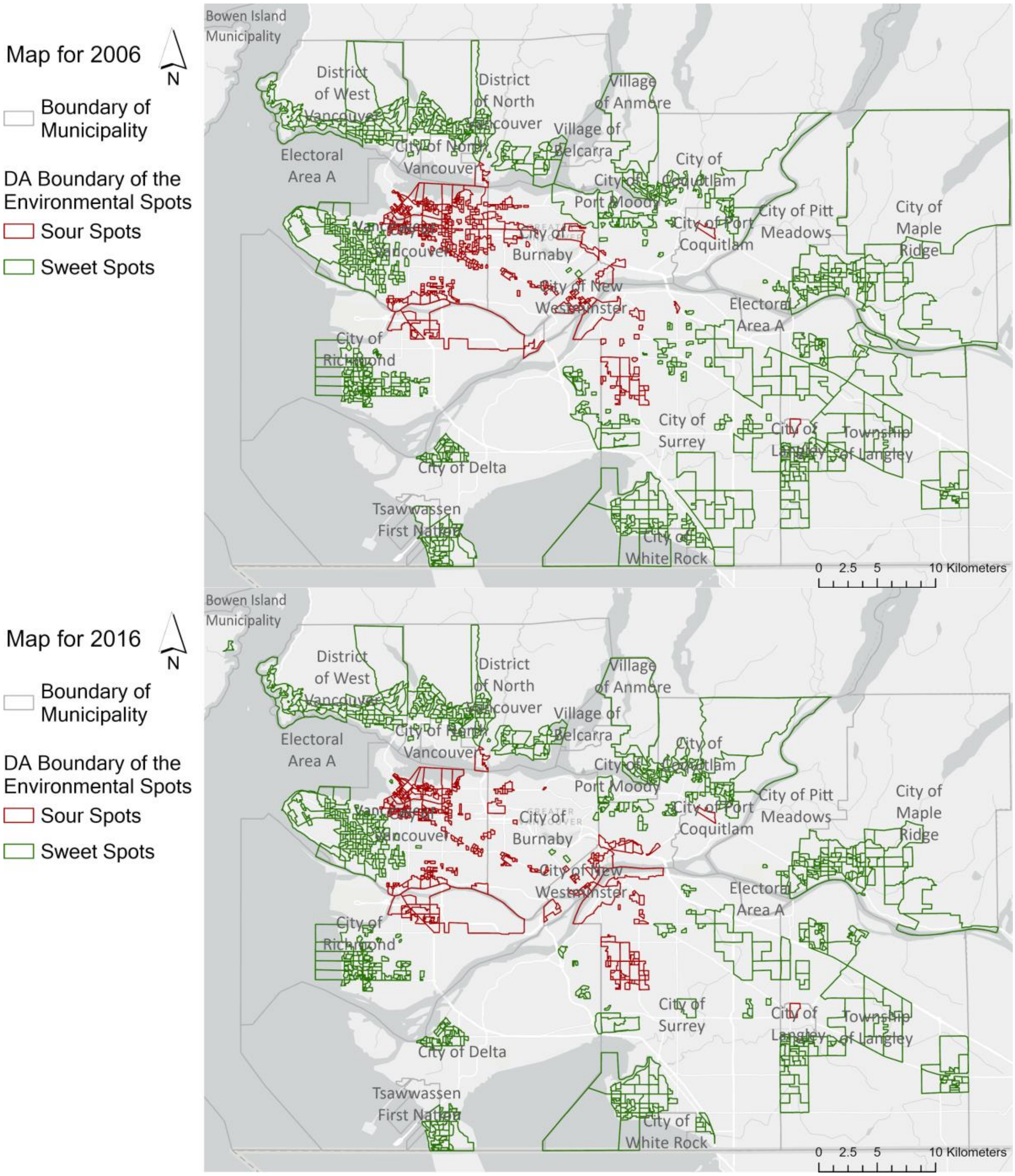

Figure S12. Spatial Distribution of Sweet and Sour Spots for Vegetation Coverage & NO_2 in 2006 and 2016.

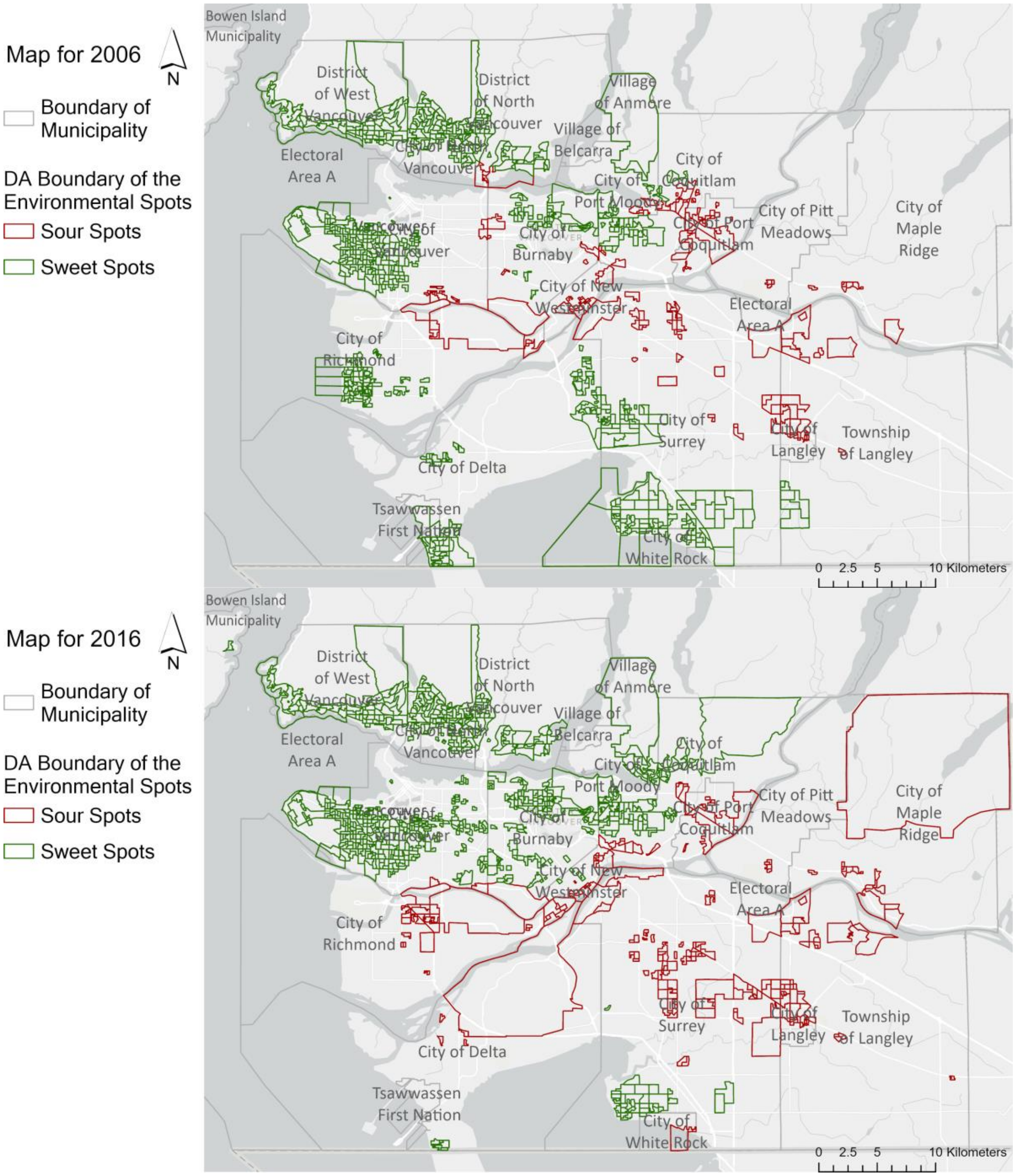

Figure S13. Spatial Distribution of Sweet and Sour Spots for Vegetation Coverage & Heat Stress in 2006 and 2016.

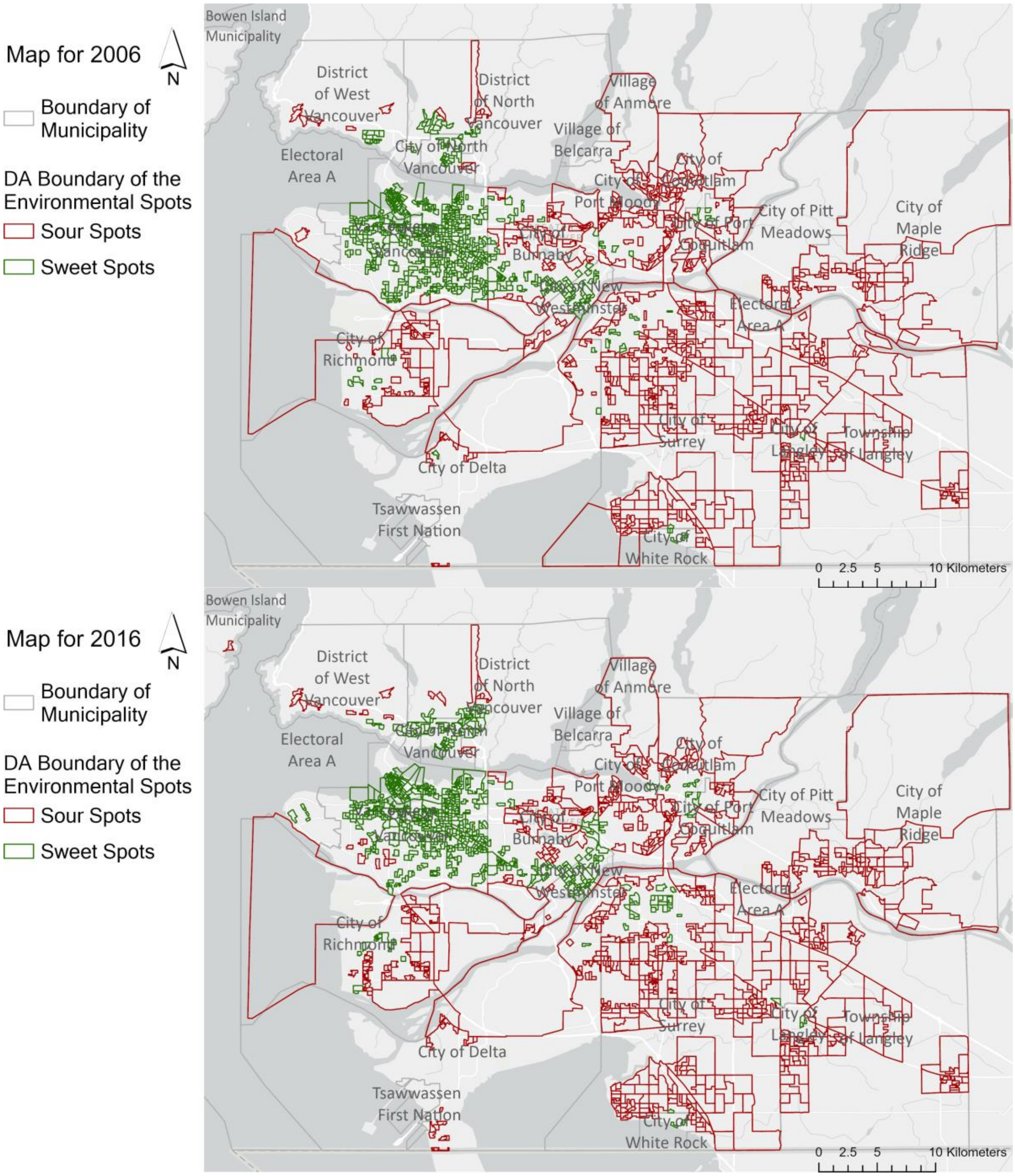

Figure S14. Spatial Distribution of Sweet and Sour Spots for Walkability & Accessibility to Park and Waterbody in 2006 and 2016.

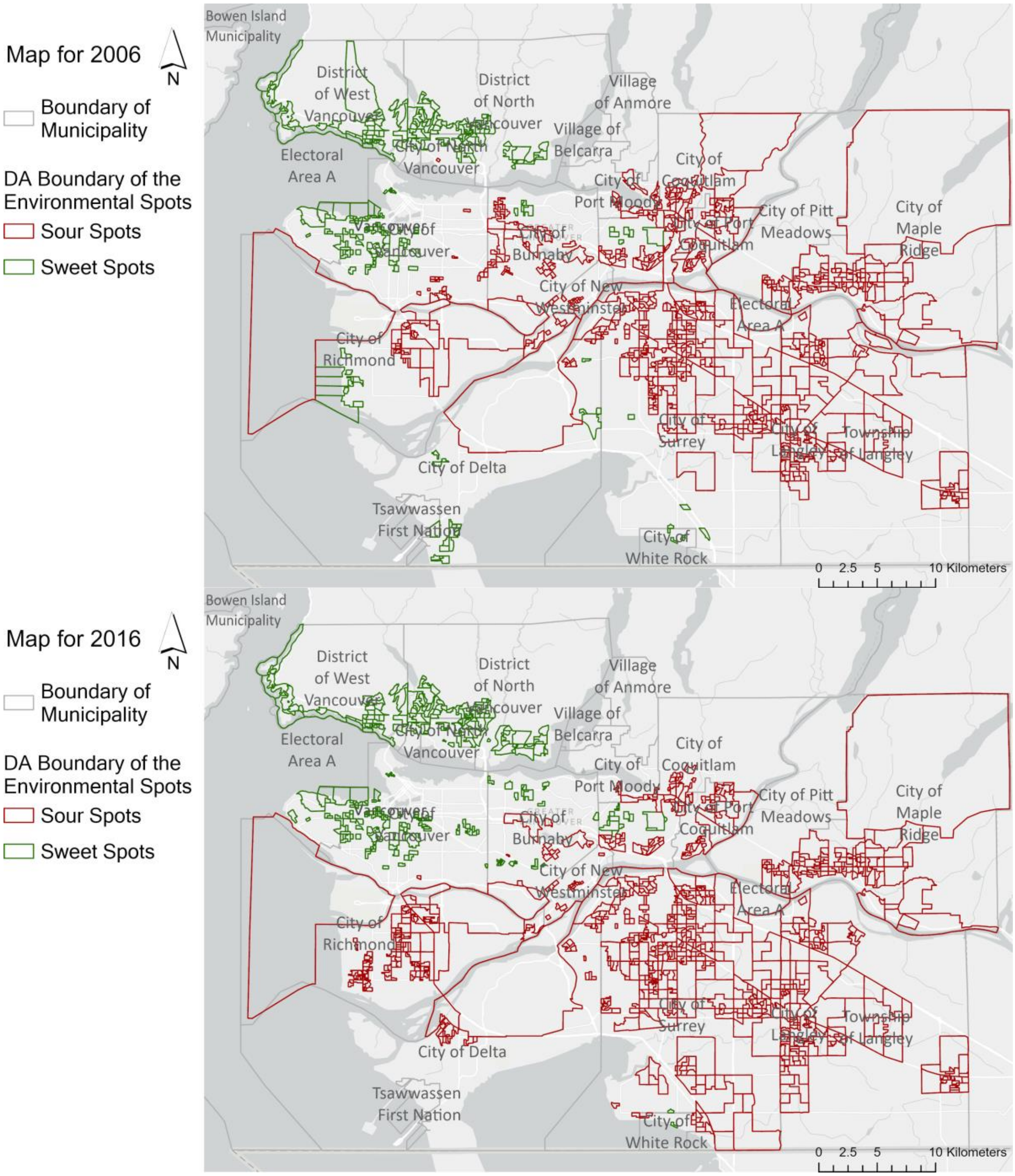

Figure S15. Spatial Distribution of Sweet and Sour Spots for Heat Stress & Accessibility to Park and Waterbody in 2006 and 2016.

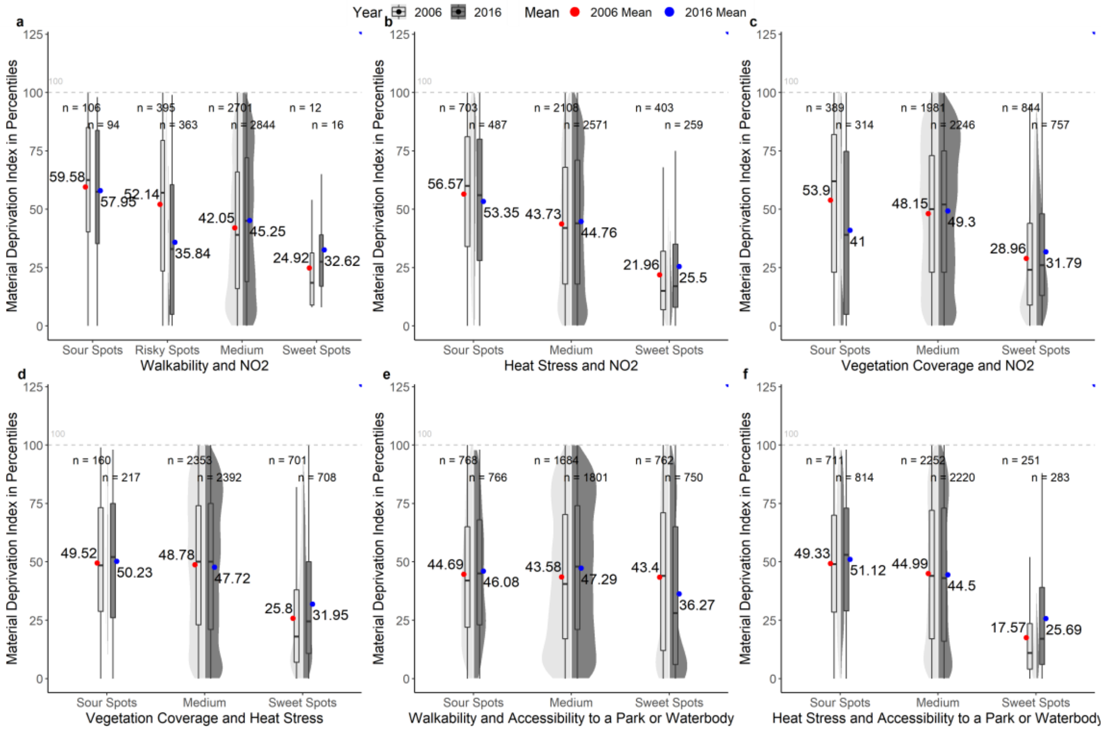

Figure S16. Statistical Summary for Material Deprivation in Different Environmental Spots

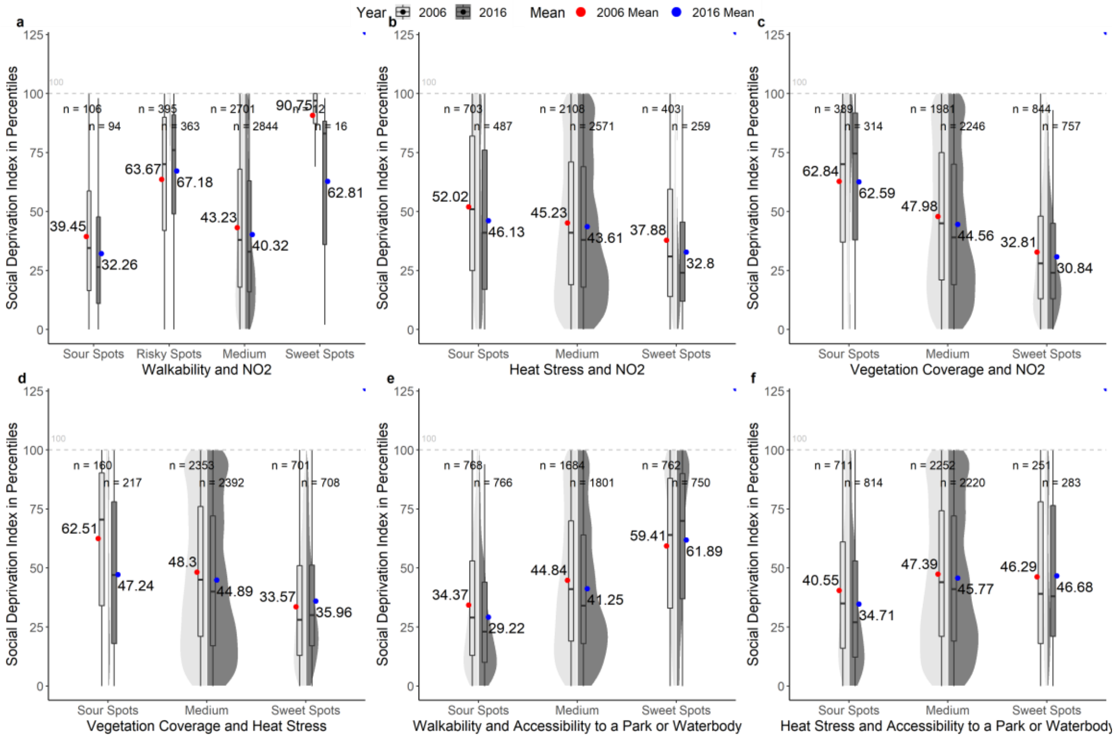

Figure S17. Statistical Summary for Social Deprivation in Different Environmental Spots

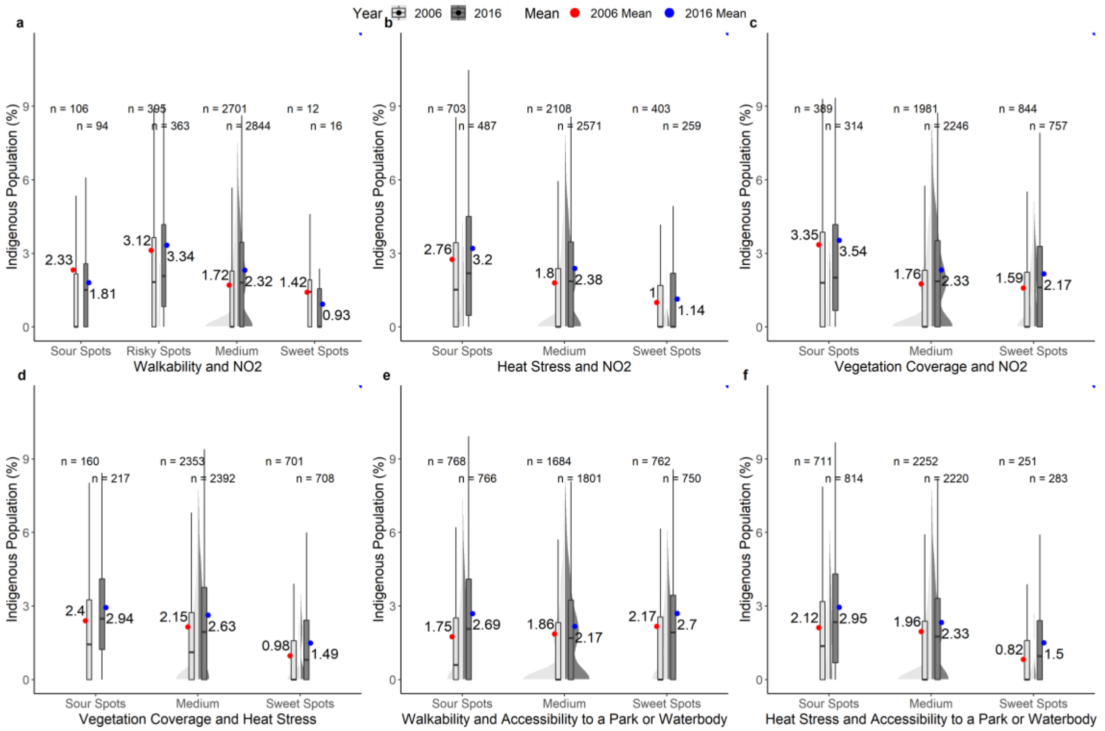

Figure S18. Statistical Summary for Indigenous Population in Different Environmental Spots

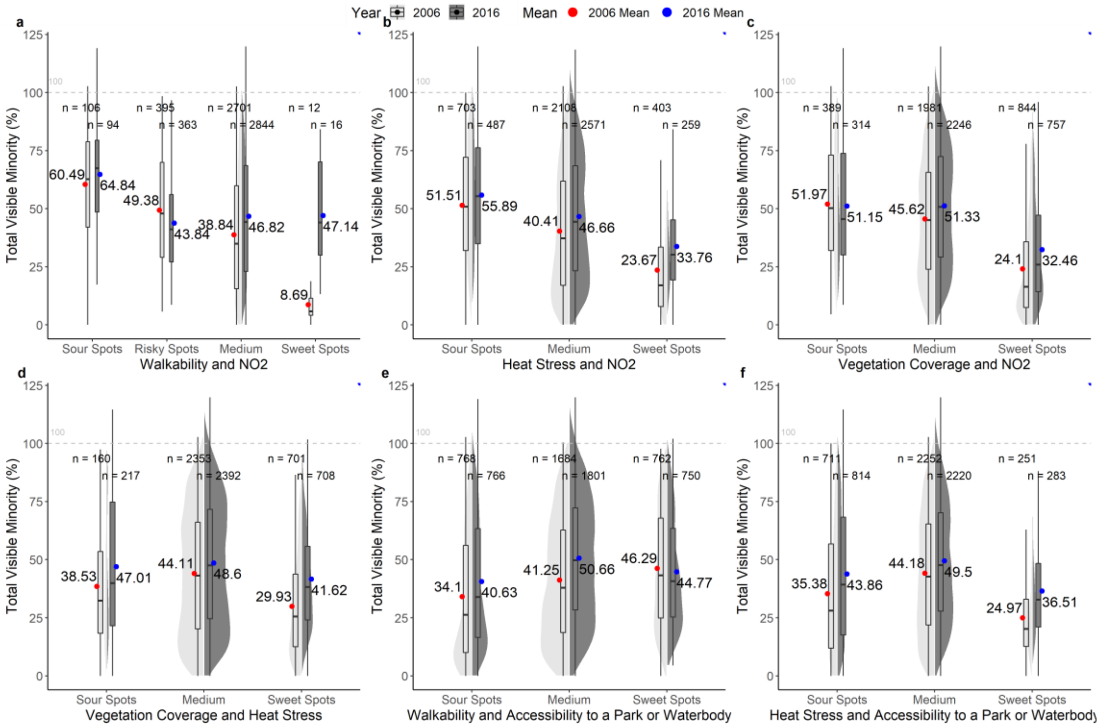

Figure S19. Statistical Summary for Total Visible Minority in Different Environmental Spots

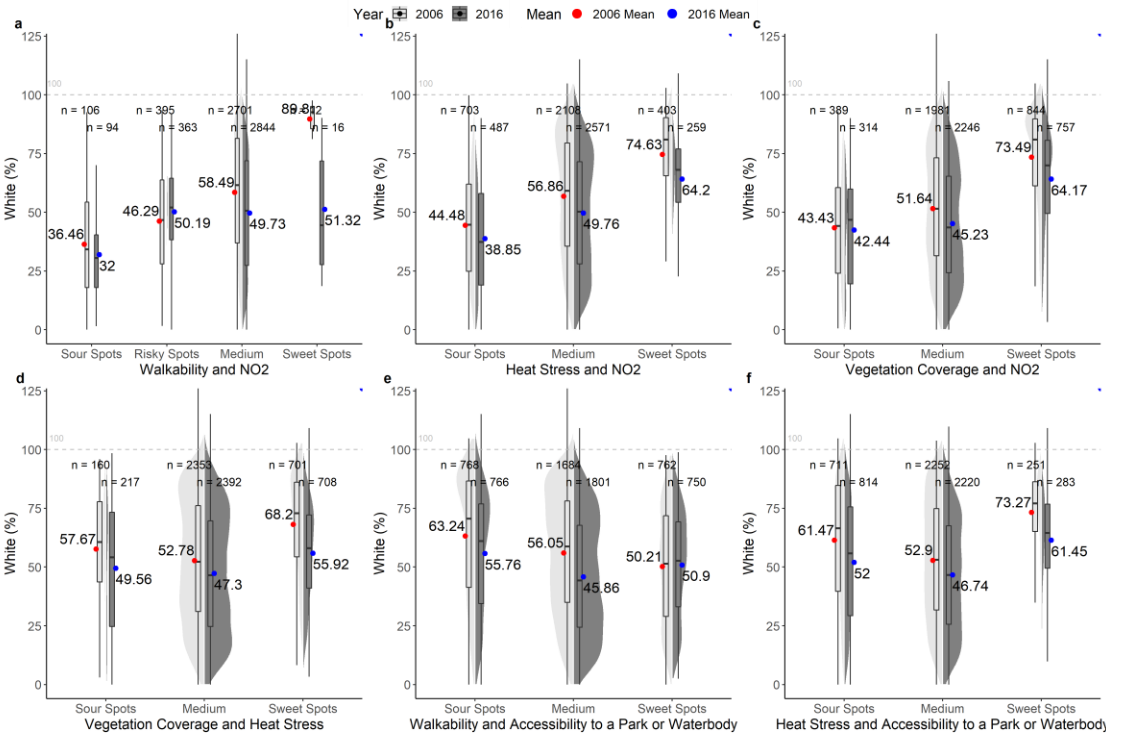

Figure S20. Statistical Summary for White Population in Different Environmental Spots

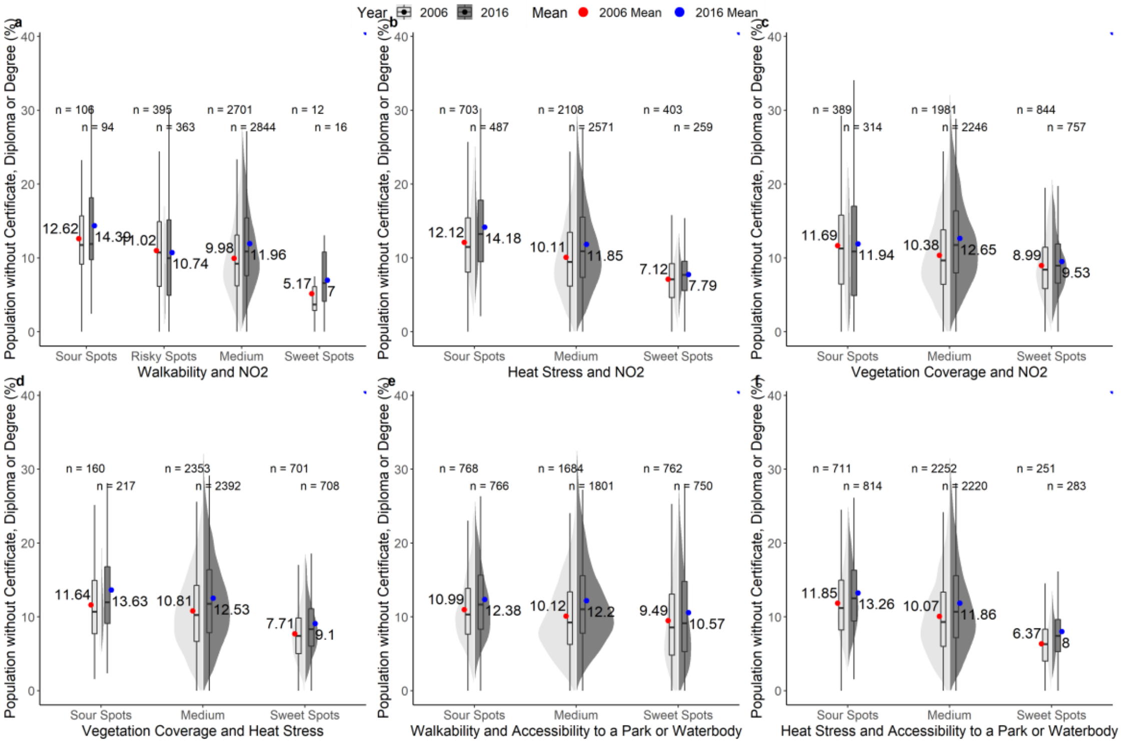

Figure S21. Statistical Summary for Population Without Higher Education in Different Environmental Spots

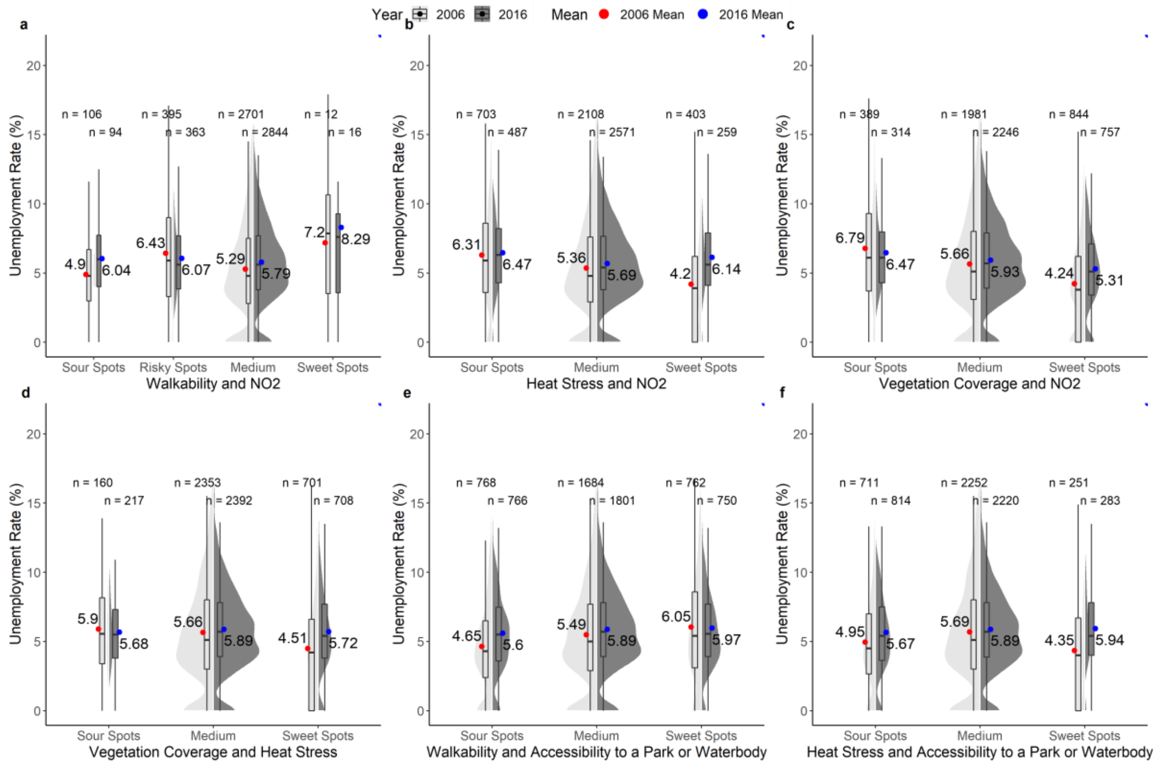

Figure S22. Statistical Summary for Unemployment Rate in Different Environmental Spots

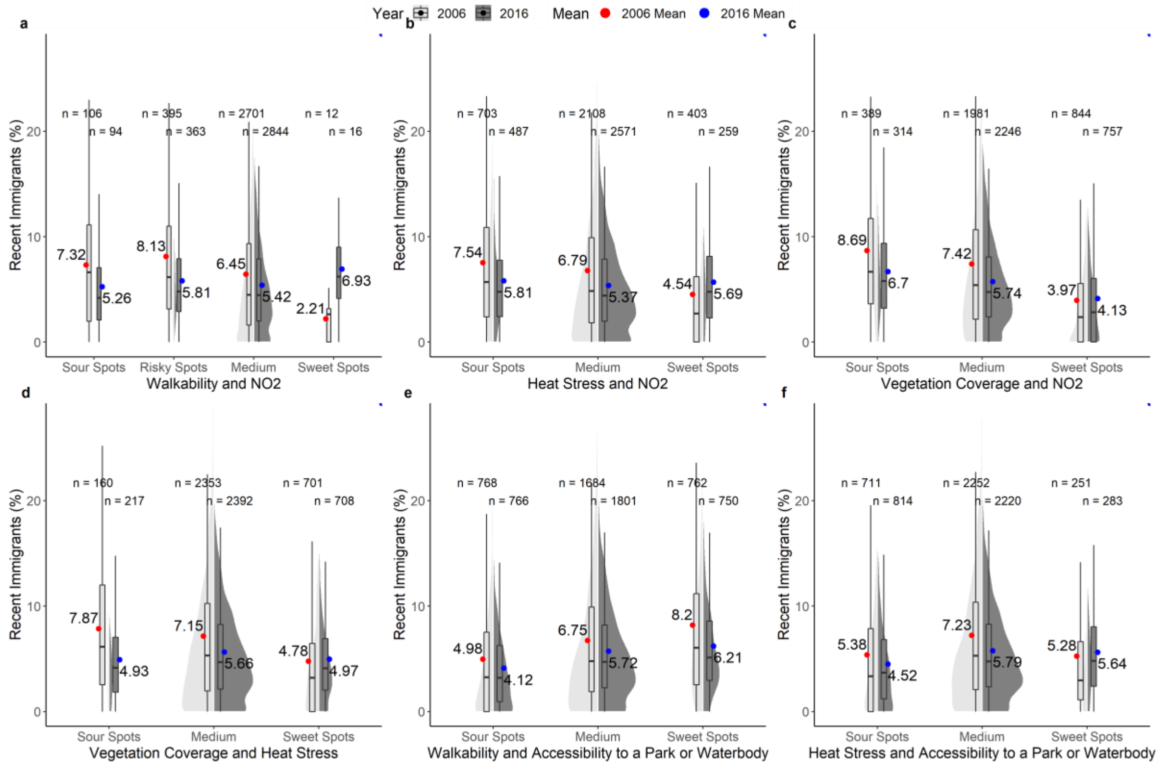

Figure S23. Statistical Summary for Recent Immigrants in Different Environmental Spots

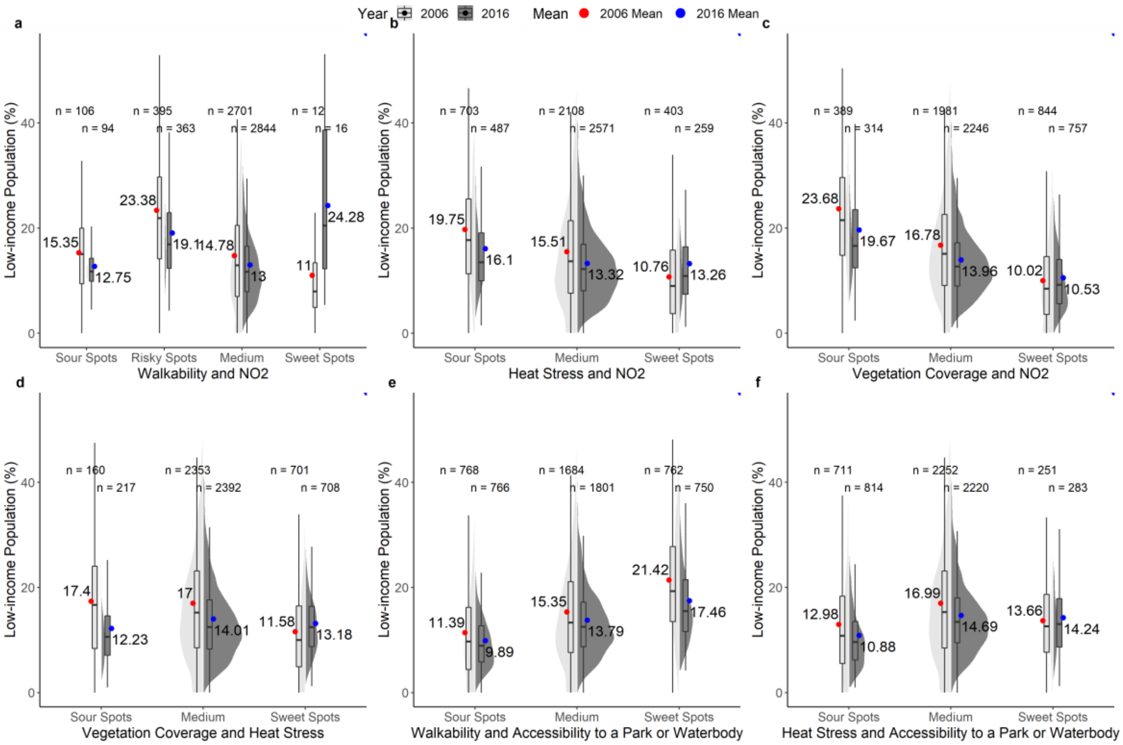

Figure S24. Statistical Summary for LICO in Different Environmental Spots

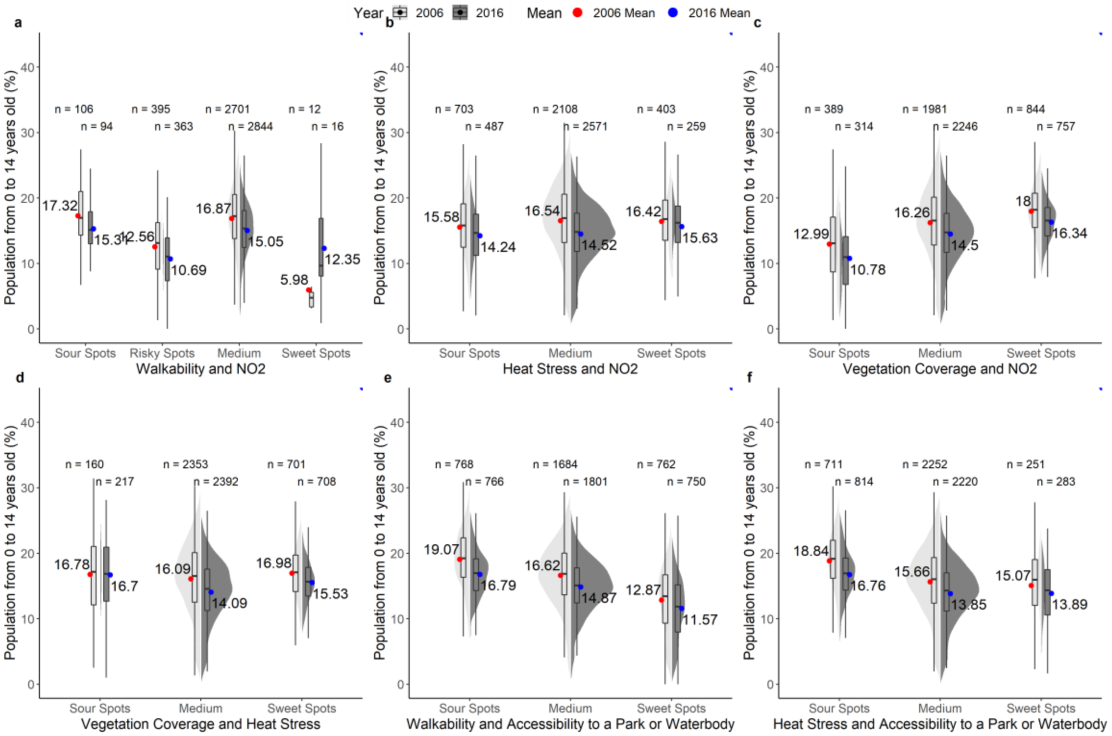

Figure S25. Statistical Summary for Population from 0 -14 Years Old in Different Environmental Spots

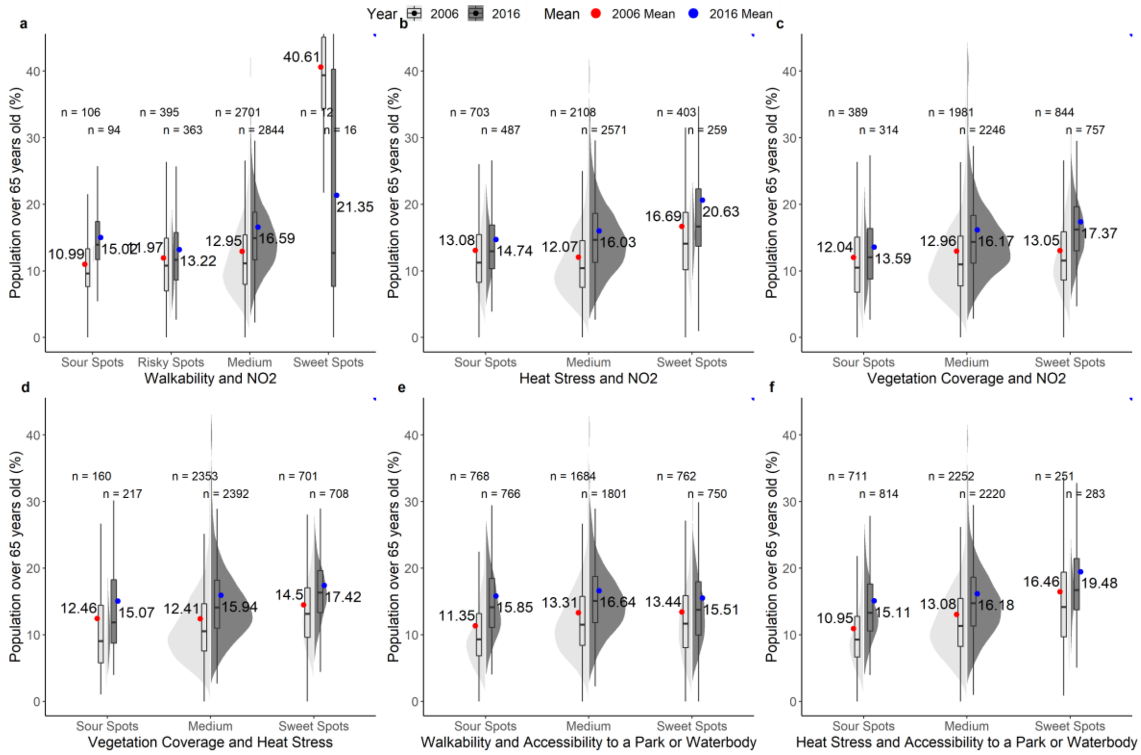

Figure S26. Statistical Summary for Population over 65 Years Old in Different Environmental Spots

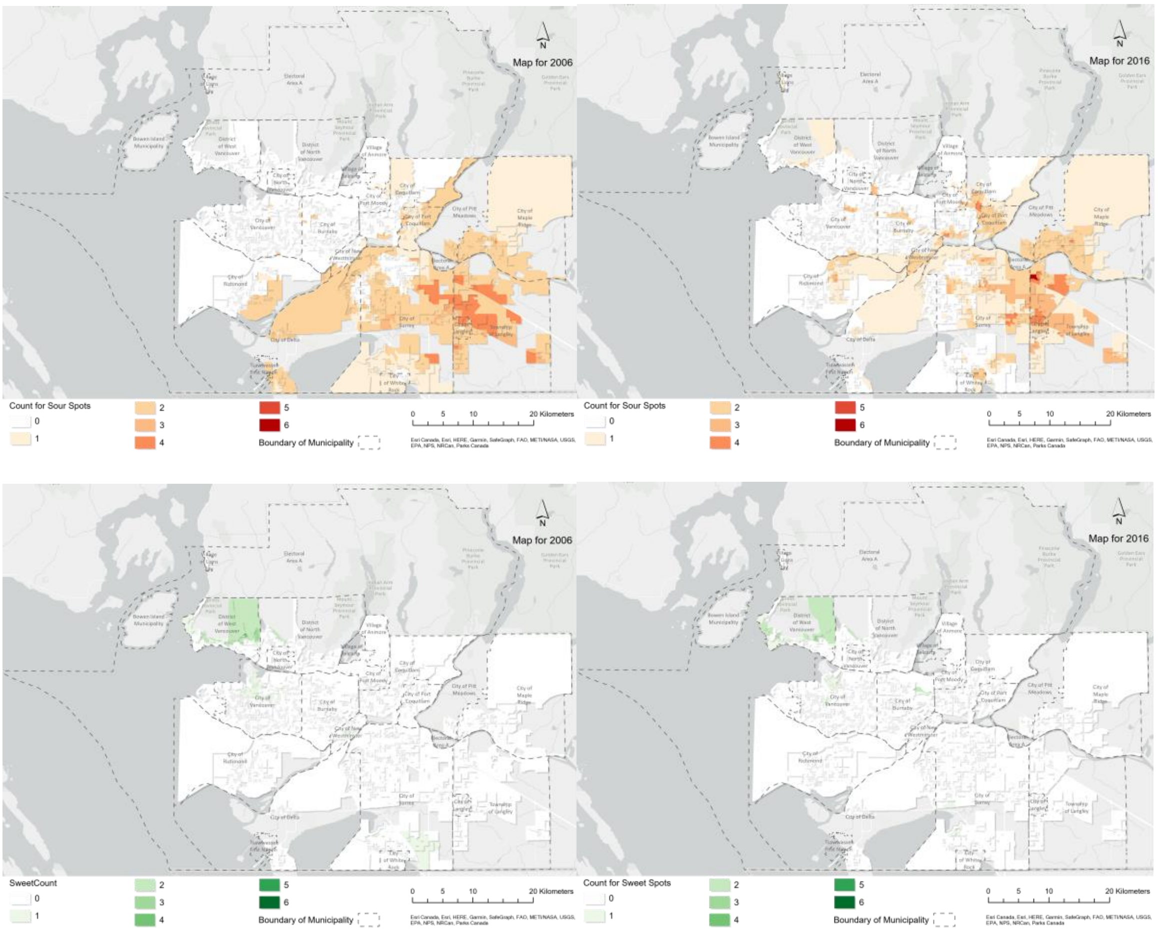

Figure S27. Sweet and sour heat maps for 2006 and 2016 indicating the total number that each DA recognized as sweet or sour spots in 6 environmental combinations from even breaks method.

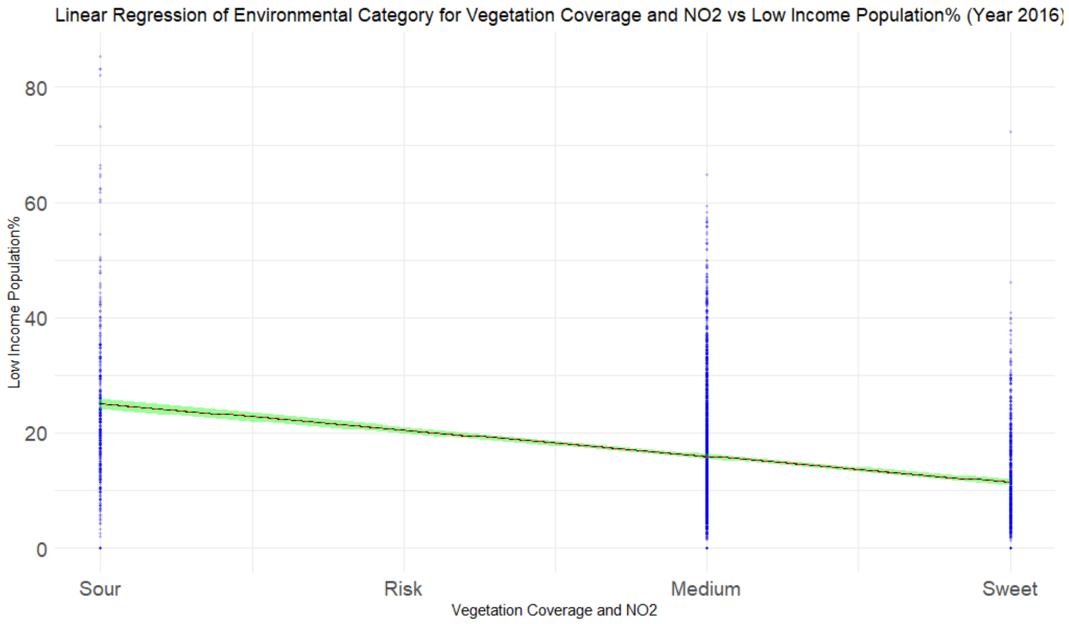

Figure S28. The Linear Regression of Environmental Category for Vegetation Coverage and NO_2 vs Low-Income Population Percentage for the Year 2016. The trend line (in red) with a 95% CI (in green) shows a slight decrease in the low-income population percentage as environmental conditions improve (moving from Sour to Sweet), which alternatively captures the differences between mean low-income percentages in different environmental categories (19.67, 13.96, and 10.53 for Sour, Medium, and Sweet Spots, respectively).

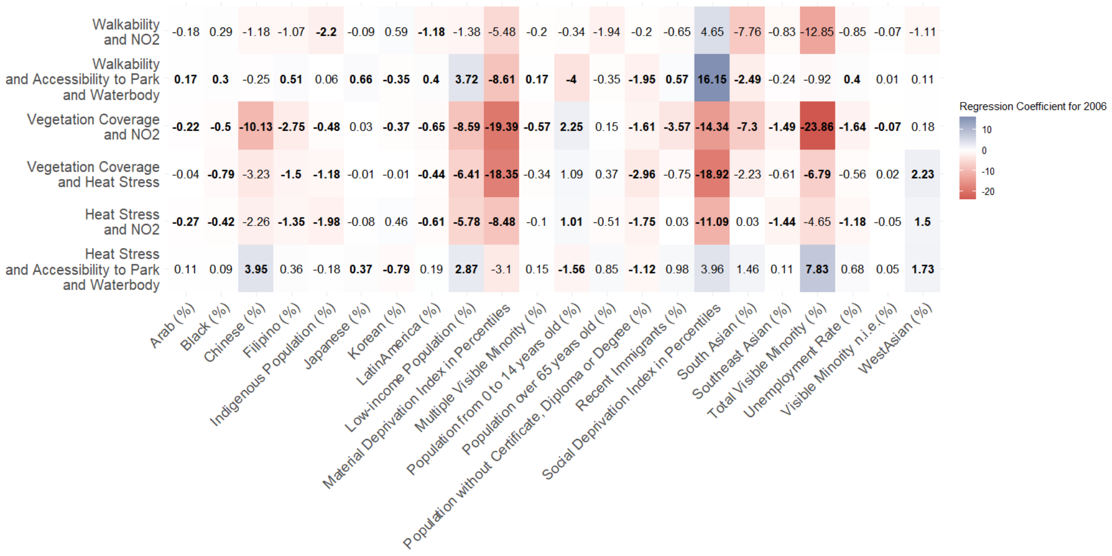

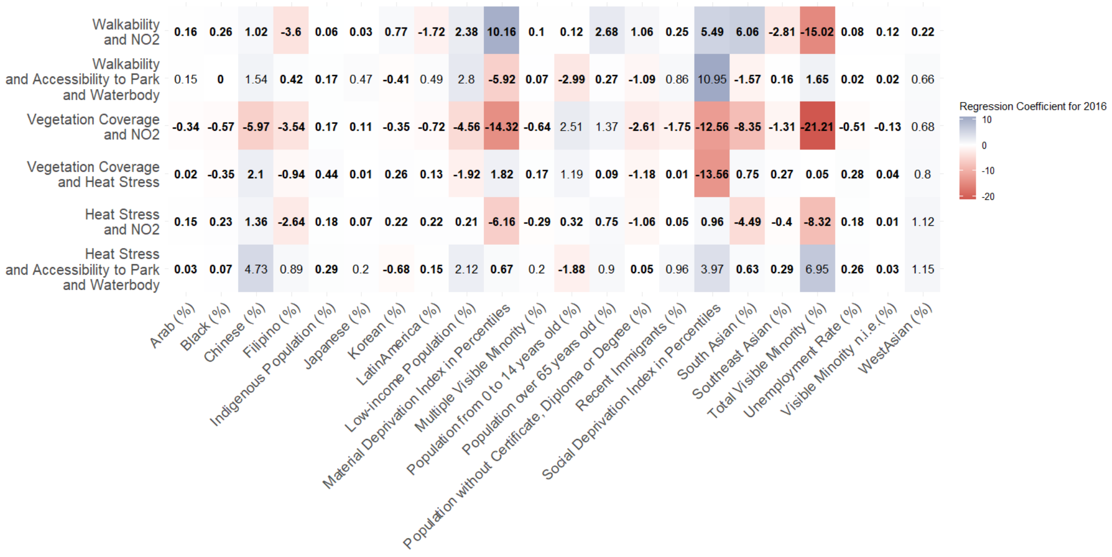

Figure S29. Heat Map for Simple Linear Regression Coefficients among Demographic Groups for Environmental Combinations in 2006 and 2016 (Even Break Method). We define inequity as a negative linear relationship between the marginalized demographic group percentage and the environmental category, indicated by a negative regression coefficient in red. Blue indicates no inequity identified for the given marginalized groups resulting from the positive a linear relationship between demographic percentage and environmental category. Numbers in bold indicate that the coefficients are statistically significant; on the contrary, numbers that are not in bold (regular font) suggest no statistically significant inequity was identified, indicated by regression coefficient with a p-value exceeding 0.05

•

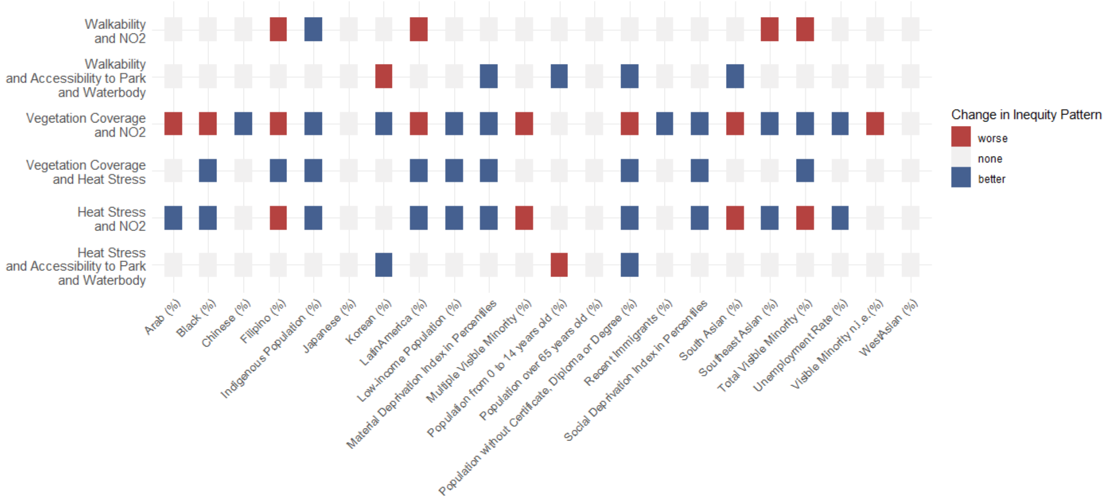

Figure S30. Changes in Distributional Environmental Inequity Patterns between the Years 2006 and 2016 from the Even Break Method. Blue indicates decreased disproportionality for marginalized groups, and red indicates increased disproportionality. Grey represents no changes identified from this method.

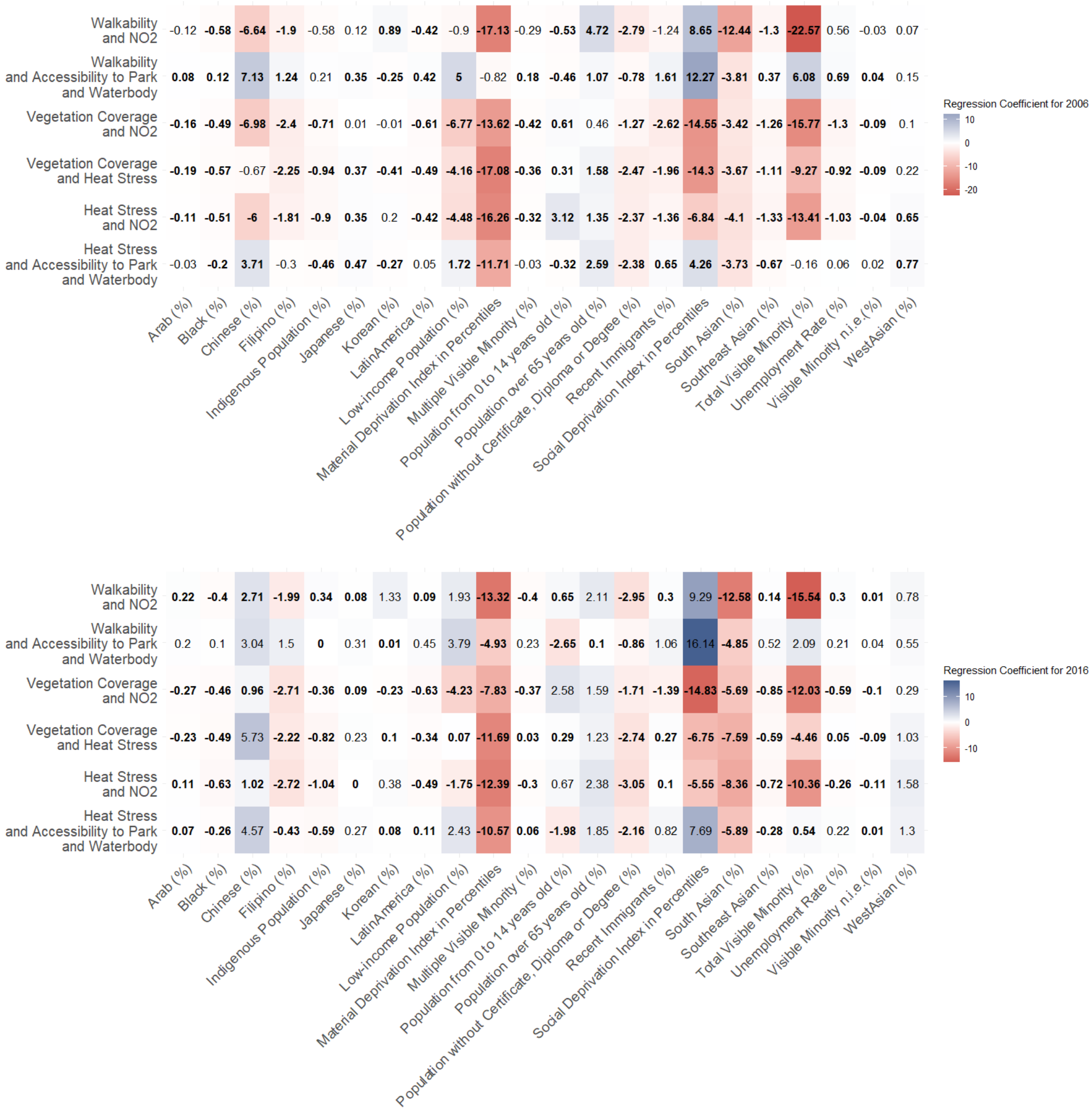

Figure S31. Heat Map for Simple Linear Regression Coefficients among Demographic Groups for Environmental Combinations without Risky Spots in 2006 and 2016 for Sensitivity Test. We define inequity as a negative linear relationship between the marginalized demographic group percentage and the environmental category, indicated by a negative regression coefficient in red. Blue indicates no inequity identified for the given marginalized groups resulting from the positive a linear relationship between demographic percentage and environmental category. Numbers in bold indicate that the coefficients are statistically significant; on the contrary, numbers that are not in bold (regular font) suggest no statistically significant inequity was identified, indicated by regression coefficient with a p-value exceeding 0.05.

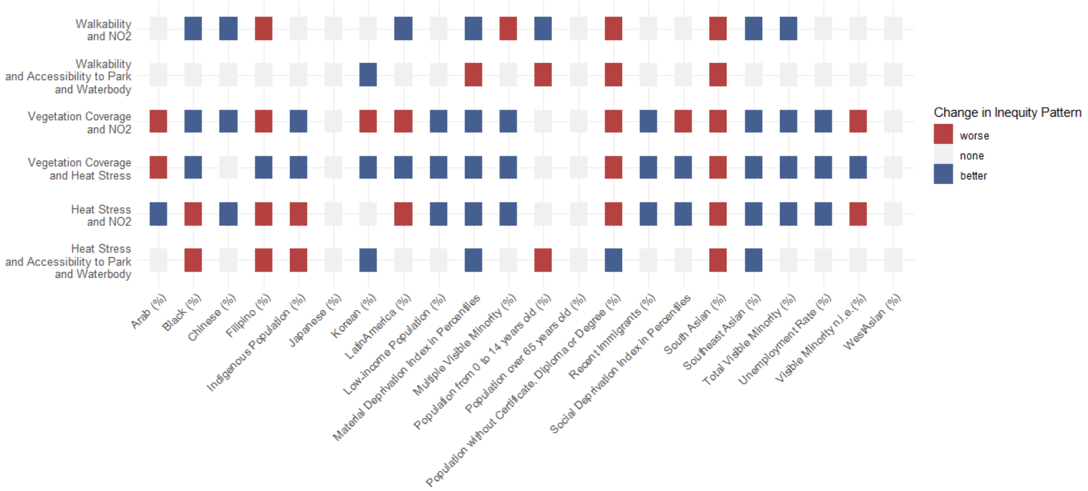

Figure S32. Changes in Distributional Environmental Inequity Patterns between the Years 2006 and 2016 from the Regression without Risky Spots for Sensitivity Test. Blue indicates decreased disproportionality for marginalized groups, and red indicates increased disproportionality. Grey represents no changes identified from this method.

Text S4. Discussion about the robustness and differences based on the sensitivity test

In order to compare the relative linear relationship between all combinations, including the one with risky spots, the independent variable needs to be the same with the same intervals. As a result, we still use 1, 2, 3, and 4 for all the regressions (in this case, some combinations don't have data points where the independent variable equals 2). Recognizing that this may artificially alter the direction and magnitude of the slope and result in different conclusions for inequity identification, we conducted a sensitivity test where we calculated regression coefficients with only categories sour, medium, and sweet, with the integer 1, 2, and 3. The results are shown in SI Figures S31 and S32. We find that the results are largely robust for most of the combinations without risky spots for inequity identification and comparison. Most of the inconsistencies in the direction of the slope are from significantly positive value (no inequity identified) to non-significant value, which are both interpreted as no sign of inequity. Only one combination changed from significant negative (inequity) to significant positive value (no inequity identified) in 2006, and two combinations in 2016. The magnitude of the slope changed, but the relative magnitude did not change; the materially and socially deprived groups and visible minorities are still the three groups that have the highest level of inequity. However, in Figure 6, the heatmap for changes between years, there are some inconsistencies resulting from regression without risky spots and regression with risky spots. Each environmental combination has discrepancies for one or two demographic groups (where regression without risky spots is identified as exacerbated inequity, but regression with risky spots is identified as improved inequity), including Population from 0 – 14 and population without higher education for walkability and accessibility to a park or water body, Lattin American and visible minority n.i.e for vegetation and heat stress,

and Filipino and Population from 0 – 14 for heat stress and accessibility to a park or water body.
